# Hydrogen peroxide inducible clone-5 sustains NADPH oxidase-dependent reactive oxygen species-c-jun N-terminal kinase signaling in hepatocellular carcinoma

**DOI:** 10.1038/s41389-019-0149-8

**Published:** 2019-08-06

**Authors:** Jia-Ru Wu, Ren-In You, Chi-Tan Hu, Chuan-Chu Cheng, Rudy Rudy, Wen-Sheng Wu

**Affiliations:** 1 Department of Molecular Biology and Human Genetics, Hualien, Taiwan; 2 Department of Laboratory Medicine and Biotechnology, College of Medicine, Tzu Chi University, Hualien, Taiwan; 3 Division of Gastroenterology, Department of Medicine, Buddhist Tzu Chi General Hospital and Tzu Chi University, Hualien, Taiwan; 4 Research Centre for Hepatology, Buddhist Tzu Chi General Hospital, Hualien, Taiwan; 5 Institute of Medical Sciences, Tzu Chi University, Hualien, Taiwan

**Keywords:** Cancer, Molecular biology

## Abstract

Target therapy aiming at critical molecules within the metastatic signal pathways is essential for prevention of hepatocellular carcinoma (HCC) progression. Hic-5 (hydrogen peroxide inducible clone-5) which belongs to the paxillin superfamily, can be stimulated by a lot of metastatic factors, such as transforming growth factor (TGF-β), hepatocyte growth factor (HGF), and reactive oxygen species (ROS). Previous studies implicated Hic-5 cross-talks with the ROS-c-jun N-terminal kinase (JNK) signal cascade in a positive feedback manner. In this report, we addressed this issue in a comprehensive manner. By RNA interference and ectopic Hic-5 expression, we demonstrated Hic-5 was essential for activation of NADPH oxidase and ROS generation leading to activation of downstream JNK and c-jun transcription factor. This was initiated by interaction of Hic-5 with the regulator and adaptor of NADPH oxidase, Rac1 and Traf4, respectively, which may further phosphorylate the nonreceptor tyrosine kinase Pyk2 at Tyr881. On the other hand, promoter activity assay coupled with deletion mapping and site directed mutagenesis strategies demonstrated the distal c-jun and AP4 putative binding regions (943–1126 bp upstream of translational start site) were required for transcriptional activation of Hic-5. Thus Hic-5 was both downstream and upstream of NADPH oxidase-ROS-JNK-c-jun cascade. This signal circuit was essential for regulating the expression of epithelial mesenchymal transition (EMT) factors, such as Snail, Zeb1, E-cadherin, and matrix metalloproteinase 9, involved in HCC cell migration and metastasis. Due to the limited expression of Hic-5 in normal tissue, it can be a promising therapeutic target for preventing HCC metastasis.

## Introduction

Hepatocellular carcinoma (HCC) is one of the most common causes of death from cancer worldwide. The poor prognosis of HCC is due to high recurrence rate mainly caused by intrahepatic metastasis (about 80%) or extrahepatic metastasis (about 20%)^1^. Tumor metastasis occurs via complicated processes, including epithelial mesenchymal transition (EMT), migration and invasion of primary tumor, followed by intravasation, extravasation, and colonization at the metastatic loci. The tumor microenvironment in HCC contains a lot of metastatic factors, such as hepatocyte growth factor (HGF)^2^ and transforming growth factorβ (TGFβ)^3^, capable of triggering HCC progression via a lot of molecular pathways. Target therapy aiming at critical signal molecules within these pathways is essential for prevention of HCC metastasis.

Recently, Hic-5 (hydrogen peroxide inducible clone-5) which belongs to the paxillin superfamily is emerging as a potential target along the metastatic signaling pathway. Hic-5 expression can be stimulated by TGF-β^4^, HGF^5^, and reactive oxygen species (ROS)^6^. Previous report demonstrated TGF-β induced Hic-5 expression to promote extracellular matrix degradation and invasion of MCF10A breast cancer cells^7^. Recent study indicated Hic-5 can regulate EMT in ovarian cancer cells in a TGFβ1-independent manner^8^. Our study revealed that Hic-5 could be induced by HGF responsible for HCC progression and may serve as a potential HCC prognosis marker^5^. In addition, Hic-5 may cooperate with paxillin to regulate metastasis of breast cancer^9^. In the tumor microenvironment, Hic-5 highly expresses in cancer associated fibroblast required for deposition and remodeling of the stromal extracellular matrix (ECM) to promote non-cell autonomous breast tumor progression^10^.

The molecular mechanisms for Hic-5 to trigger EMT and tumor progression appeared to be closely associated with ROS signaling. Not only that Hic-5 gene expression can be induced by reactive oxygen species (ROS)^11^, as its name suggest, it also has a great impact on ROS generation. Previously, Hic-5 was found to participate ROS generation during endothelial cell migration^11^. In this context, Hic-5 serves as an adaptor for assembling focal adhesion complex required for NADP oxidase-dependent ROS production^11^. In the vascular smooth muscle cells, Hic-5 mediated TGFβ-induced activation of NADPH oxidase required for ROS generation and cell adhesion^12^. Recently, we also found Hic-5 regulated the ROS-c-jun N-terminal kinase (JNK) signaling pathway for HCC progression^5^. Notably, Hic-5 seems to locate both upstream and downstream of ROS-JNK cascade. However, whether and how Hic-5 cross-talks with the ROS-JNK cascade in a positive feedback manner was not fully elucidated. In this report, we address this issue in a comprehensive manner, demonstrating that Hic-5 interacted with the regulators of NADPH oxidase, including Rac-1, Traf4, and Pyk2, for activating NADPH oxidase-ROS-JNK-c-jun cascade, which in turn was required for Hic-5 transcriptional activation. This positive feedback circuit was essential for elevating mesenchymal transcriptional factors, such as Snail, Zeb1 and the matrix degradation enzyme, matrix metalloproteinases 9 (MMP-9) and decreasing the epithelial marker E-cadherin, involved in HCC cell migration and metastasis.

## Results

### Hic-5 was required for activation of ROS-JNK-c-jun cascade and elevation of mesenchymal markers

**Fig. 1:**
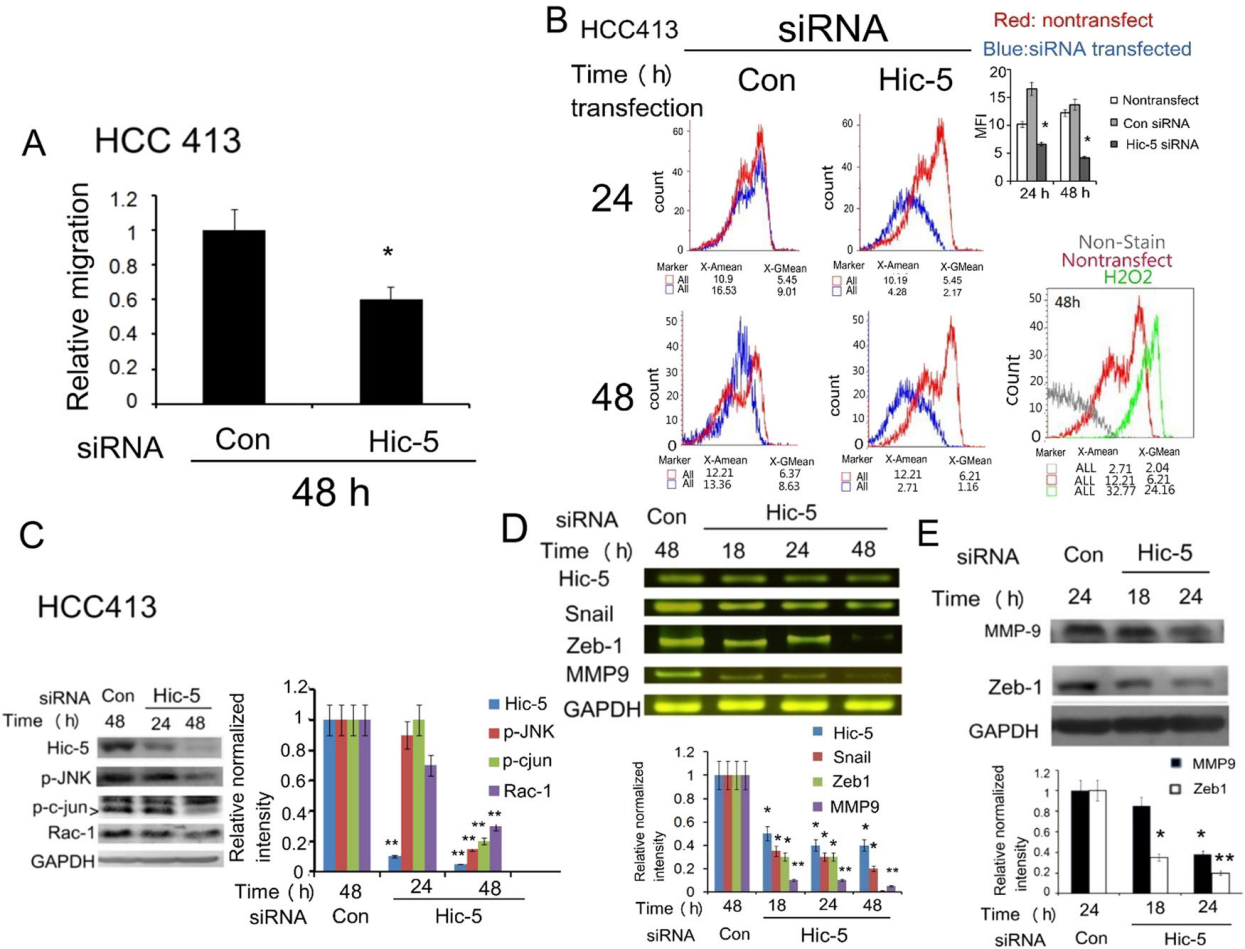
Depletion of Hic-5 suppressed ROS generation, phosphorylation of JNK/c-jun and expression of Rac-1, Snail, and Zeb-1 and cell migration. HCC413 were untransfected (non) or transfected with Control (Con) or Hic-5 siRNA for indicated time. Transwell migration (**a**) DCF-DA ROS generation assays (**b**), western blot of indicated molecules (**c**) and (**d**) and RT/PCR of indicated molecules (**d**) were performed. In (**a**), relative migration was calculated as the ratio of the intensity (scanned by ImageJ software) of migrated cells from that transfected with Hic-5 vs that with control (Con), taking the data of control as 1.0. In (**b**), curve plots overlaying non-transfected (red curves) and control siRNA or Hic-5 siRNA- transfected groups (blue curves) are shown with the mean fluorescence intensity (MFI) values below (left panel). Quantitative histogram of MFI for comparison of control siRNA and Hic-5 siRNA at each time point was shown in the right higher panel. (*) represent the statistically significant difference (*p* < 0.05) between Hic-5 siRNA and control siRNA group. In the right lower panel of (**b**), the curves of unstained cell and H_2_O_2_-treated cell (48 h) overlapping with that of untreated cell are shown as negative and positive controls, respectively. In (**c**), (**d**) and (**e**), the quantitative figures were shown in right (**c**) or lower (**d**) and (**e**) panel. The intensity of each molecule was normalized with GAPDH. Relatively normalized intensity was calculated taking the data of control siRNA (Con si) group as 1.0. Each data was average of five reproducible experiments with coefficient of variation (C.V.) 9.5%. (**, *) represent the statistically significant difference (*p* < 0.005, *p* < 0.05, respectively) between the indicated Hic-5 siRNA with control siRNA group of the indicated molecules

We have proposed Hic-5 mediated ROS-JNK signal cascade in a positive feedback manner in HCC329^5^. To validate whether this is a general pathway for triggering HCC progression, additional patient-derived cells with higher metastatic potential such as HCC413 were employed. Initially, we investigated whether Hic-5 was required for migration and invasion of HCC413. As demonstrated in Fig. 1a, depletion of Hic-5 by transfection of Hic-5 siRNA for 48 h decreased cell migration of HCC413 (using transwell migration assay) by about 44% as compared with that of the cells transfected with control siRNA. The decrease of motility of Hic-5-depleted HCC413 is in consistent with that observed in our previous report using HCC329^5^. Moreover, cell invasion (using matrigel-coated transwell assay) of HCC413 transfected with Hic-5 siRNA decreased by 85% compared with the control siRNA transfected cell (Supplementary Fig. 1, upper panel). On the molecular level, ROS generation using DCF-DA (specific for detecting H_2_O_2_) was suppressed in HCC413 transfected with Hic-5 siRNA by 66% compared with the cells with control siRNA (Fig. 1b). Furthermore, phosphorylation of JNK (Fig. 1c) was decreased by 80% in HCC413 transfected Hic-5 siRNA for 48 h compared with that in the control siRNA group (*p* = 0.004, *N* = 5). The decrease of phosphorylated JNK in Hic-5 depleted HCC413 was consistent with that observed in the previous report using HCC329^5^. Moreover, phosphorylation of c-jun, which is the transcription factor downstream of JNK, was also suppressed by 90% (*p* = 0.001, *N* = 5) in Hic-5-depleted HCC413 (Fig. 1c). On the other hand, gene expression of the mesenchymal transcriptional factors such as Snail^13,14^ and Zeb1^15,16^ and one of the matrix degradation enzymes, matrix metalloproteinase 9 (MMP9), known to be involved in tumor progression, were also examined. As shown in Fig. 1d, mRNA of Snail, Zeb-1 and MMP9 significantly decreased by 45–70% in HCC413 transfected with Hic-5 siRNA (compared with those in HCC413 transfected with control siRNA) for 18–48 h in a time-dependent manner. Quantitative RT-PCR further confirmed the decrease of mRNAs of Snail and MMP9 by 50% and 53% (*p* = 0.01, *N* = 3), respectively, in HCC413 transfected with Hic-5 siRNA for 48 h (compared with those in HCC413 transfected with control siRNA) (Supplementary Fig. 2A). Western blots also demonstrated MMP9 and Zeb-1 proteins were decreased in HCC413 transfected with Hic-5 siRNA for 24 h by 70–80% compared with those of the cells transfected with control siRNA (Fig. 1e). The knockdown efficiency of Hic-5 siRNA was validated by the decrease of Hic-5 mRNA by 85% (*p* = 0.0008, *N* = 6) (Fig. 1d) and protein by 95% (*p* = 0.007, *N* = 5) (Fig. 1c) in HCC413 transfected with Hic-5 siRNA compared with those in HCC413 transfected with control siRNA.

### Hic-5 was sufficient for activating ROS-JNK signaling that regulate EMT markers and elevating HCC340 invasion

**Fig. 2:**
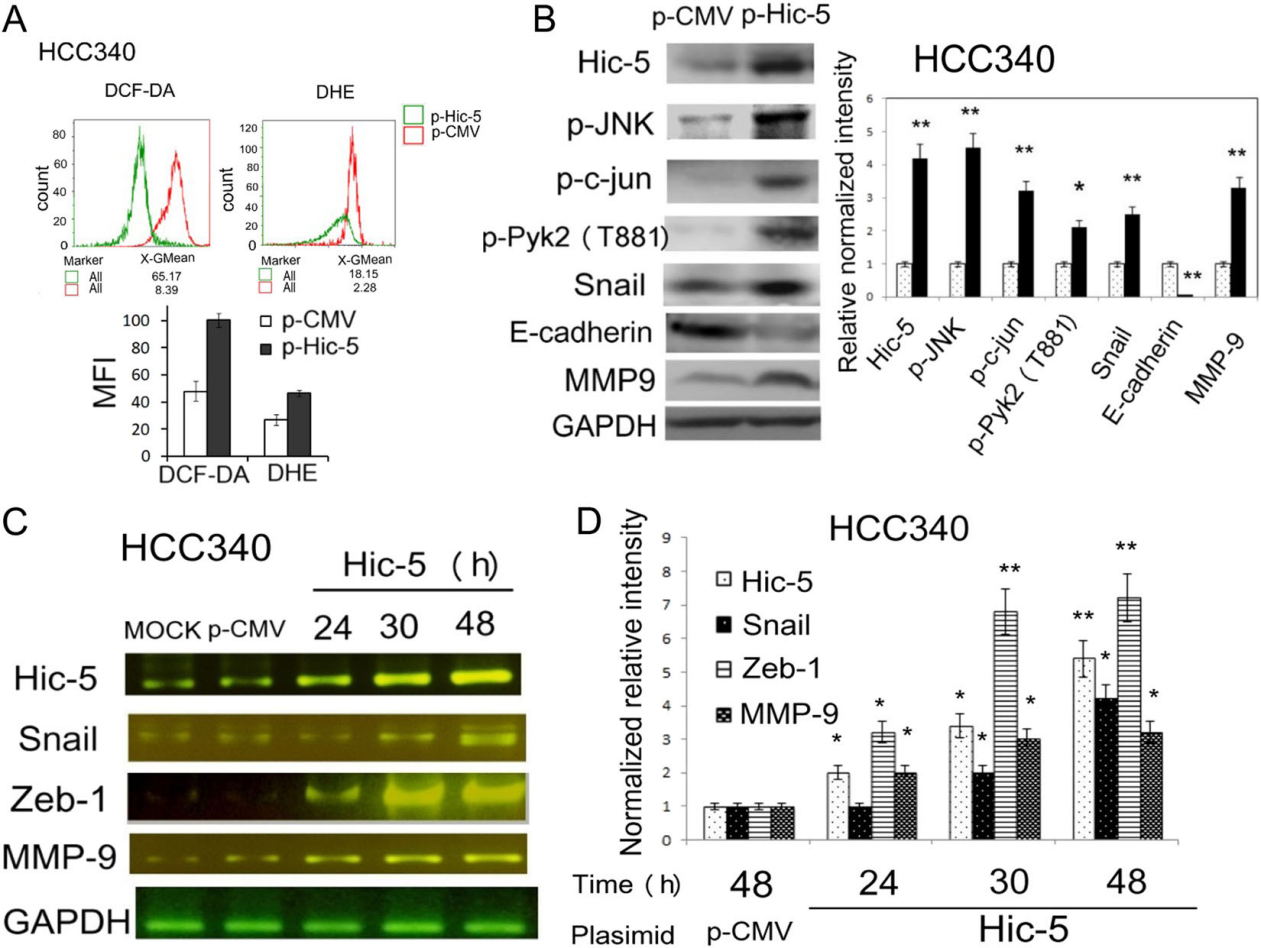
Overexpression of Hic-5 activated ROS-JNK cascade and regulated EMT markers. HCC340 were transfected with pCMV vector or Hic-5 expression plasmid (p-Hic-5) for indicated times (**a**, **c**) or 48 h (**b**), ROS generation assay (**a**), western blot (**b**) and RT/PCR (**c**) of indicated molecules were performed. In (**a**), re-suspended cells were incubated with ROS probes DCF-DA (0.2 mM) or dihydroethidium (DHE) (0.2 mM) at RT for 1 h. The fluorescence of the cell with DCF-DA or DHE were measured by flow cytometry. The indicated fluorescent intensity curve of p-CMV or p-Hic-5 group at 24 h after transfection are overlapped for comparison. Quantitation of MFI for different groups was shown in the lower panel. In (**b**), the quantitative figures of western blot were shown in right panel. Panel (**d**) is the quantitative figure of (**c**). The intensity of each molecule was normalized with GAPDH. Relative normalized intensity was calculated taking the data of control p-CMV group as 1.0. Each data was average of five reproducible experiments with C.V. of 10%. In each quantitative figure, (**, *) represent the statistically significant difference (*p* < 0.005, *p* < 0.05, respectively) between the indicated p-Hic-5 group and p-CMV group

To investigate whether enhancement of Hic-5 per se may trigger downstream ROS-JNK signaling, another patient-derived HCC cell line HCC340 with low Hic-5 expression^5^, was employed. This cell line is suitable for ectopic expression of Hic-5 followed by observing its impact on ROS-JNK-c-jun pathway. Our previous report has shown overexpression of Hic-5 increased the level of ROS generation in HCC340 using DCF-DA^5^. Further, we examined whether both H_2_O_2_ and superoxide anions (O_2_^−^) can be elevated in HCC340 overexpressing Hic-5. Thus Dihydroethidium (DHE), a probe specific for detecting O_2_^−^ was also included. As demonstrated in Fig. 2a, transfection of Hic-5 expression plasmid (p-Hic5) in HCC340 for 24 h greatly elevated ROS generations by twofold as compared with the control vector (p-CMV) group, using either DCF-DA or DHE labeling methods. These were consistent with the significant elevations (by 2.5∼4.0-fold) of phosphorylation of JNK and p-c-jun in HCC340 after transfection of p-Hic5 for 24 and 30 h (Fig. 2b). On the other hand, RT/PCR analysis demonstrated that transfection of p-Hic5 for 24–48 h increased the mRNAs of Snail, Zeb-1 and MMP9 by 3∼7-fold (Fig. 2c, d) in a time-dependent manner. Quantitative RT-PCR further confirmed the increase of mRNAs of Snail and MMP9 by 2.8 and 5.0-fold (*p* = 0.04, *N* = 3), respectively, in HCC340 overexpressing Hic-5 for 48 h compared with those in the p-CMV group (Supplementary Fig. 2B). Consistently, dramatic elevation of Snail and MMP-9 proteins coupled with decrease of E-cadherin (an epithelial marker well known to be suppressed by Snail) can be observed in HCC340 overexpressing Hic-5 (Fig. 2b). In the matrigel-coated transwell assay, we also observed invasion of HCC340 transfected with p-Hic-5 for 48 h greatly increased compared with that of p-CMV group (Supplementary Fig. 1, lower panel).

### Hic-5 is essential for activation of NADPH oxidase

**Fig. 3:**
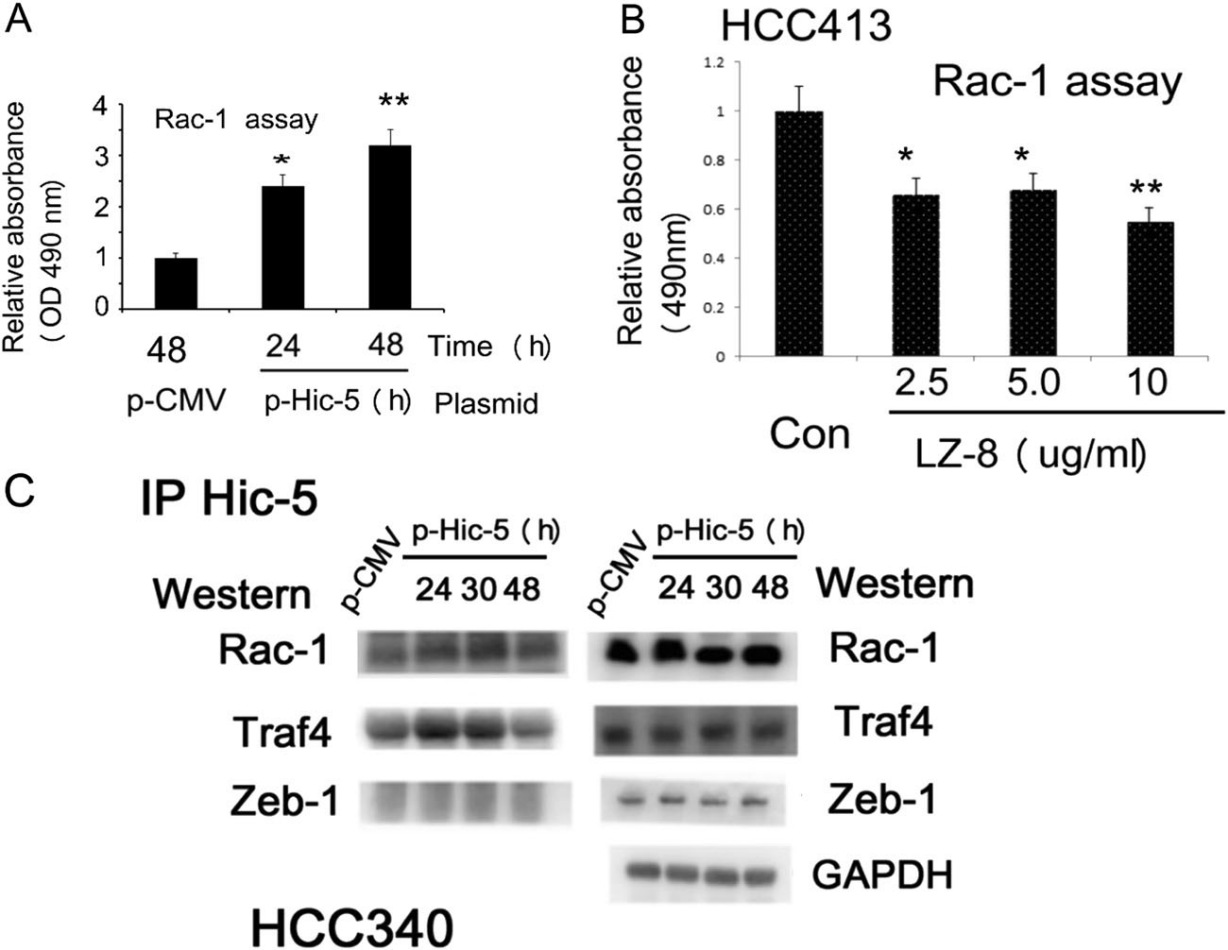
Rac-1 activity was regulated by Hic-5 and inhibited by LZ-8, and Hic-5 interact with regulators of NADPH oxidase. HCC340 cells were transfected with Hic-5 expressing plasmid (p-Hic-5) or p-CMV vector for indicated time (**a**) and (**c**), HCC413 cells were untreated (Con) or treated with 2.5-10 μg/ml LZ-8 for 24 h (**b**), the cell lysates were then harvested for Rac-1 activity (**a**), (**b**) and immunoprecipitation of Hic-5 followed by western blot of indicated molecules (**c**, left panel) or western blot of indicated molecules (**c**, right panel). In (**a**) and (**b**), relative Rac-1 activity was calculated, taking the p-CMV (**a**) or untreated (**b**) group as 1.0. The data shown are representative of three reproducible experiments with coefficient variation (C.V.) of 9.5%. (**, *) represent the statistically significant difference (*p* < 0.005, *p* < 0.05, respectively) between the indicated groups and p-CMV (**a**) or untreated (**b**) group. In (**c**), GAPDH was used as an internal control

We further explored the mechanisms by which Hic-5 triggers the ROS-JNK signal cascade. In the nonphagocytic cell NADPH oxidase is the enzyme responsible for generating ROS to mediate signal transduction for various pathophysiological processes including cancer progression^17,18^. It was well known that Rac-1 GTPase is one of the regulators of NAPDH oxidase on focal adhesion^17–19^. Interestingly, we found Rac-1 protein was significantly decreased in HCC413 depleted of Hic-5 (Fig. 1b). On the other hand, slight decrease of Rac-1 activity can be observed in Hic-5 depleted HCC413 (data not shown). Moreover, Rac-1 activity increased by threefold in HCC340 transfected with Hic-5 expressing plasmid for 48 h, compared with that in the p-CMV vector group (*p* = 0.004, *N* = 3) (Fig. 3a). In addition, Rac-1 activity in HCC413 was suppressed by 40–45% by the Chinese herbal peptide Ling-Zhi 8 (LZ-8, purified from Ganoderma lucidium) (at 2.5–10 μg/ml), which is a strong inhibitor of Hic-5^5^ (Fig. 3b). Therefore, it is very probable that Hic-5 may regulate Rac-1 for activating NADPH oxidase. Interestingly, transient knockdown of Hic-5 dramatically reduced constitutive NADPH oxidase activity in HCC413 (Supplementary Fig. 3A), and prior transfection of Hic5 siRNA significantly prevented HGF-induced NADPH oxidase activity in HCC340 at 4 and 8 h by 40–60% as compared with that in the cells transfected with control siRNA (Supplementary Fig. 3B). Moreover, overexpression of Hic-5 in HCC340 greatly elevated NADPH oxidase activity by 4∼6.5-fold (Supplementary Fig. 3C).

### Hic-5 interacts with the regulators of NADPH oxidase for activating its enzyme activity

To fully understand the mechanisms for Hic-5-triggered NADPH oxidase activation, we try to identify the Hic-5 interacting molecules critical for regulating NADPH oxidase on the focal adhesion. In addition to Rac-1, there are two candidate molecules that may interact with Hic-5 for activating NADPH oxidase. One of them is Traf4 which is a binding partner of a NADPH oxidase adaptor p47(phox) known to be associated with Hic-5 for ROS generation on the focal adhesion^11^. Also, proline rich tyrosine kinase2 (Pyk2), a nonreceptor tyrosine kinase on the focal adhesion is capable of activating NADPH oxidase-dependent ROS generation and on the contrary, activated by NADPH oxidase-dependent ROS signaling^20–22^. As shown by IP/western analysis, Hic-5 can be associated with Rac-1, Traf4, and Pyk2, but not with Snail and MMP9 (as negative controls) in HCC413 (Supplementary Fig. 4A). In addition, association of Hic-5 with Rac-1 and Pyk2 can be significantly suppressed by LZ-8 (5 μg/ml) (Supplementary Fig. 4A, B). On the other hand, associations of Hic-5 with Traf4, and Rac-1 increased by 2∼4 fold in HCC340 after transfection of Hic-5 expression plasmid for 24–30 h and declined at 48 h (Fig. 3c). As a negative control, Zeb-1 did not associate with Hic-5 at any time points, although it was significantly expressed in HCC340 (Fig. 3c). Although Pyk2 was not associated with Hic-5 in HCC340 overexpressing Hic-5 (data not shown), phosphorylation of Pyk2 (at tyrosine 881), known to be critical for EMT and cell migration^23^, was significantly elevated (Fig. 2b). Together, these results indicated Hic-5 could activate NADPH oxidase via interacting with the regulators and adaptors of NADPH oxidase in focal adhesion. To validate the role of Traf4 and Pyk2 play in the Hic-5-triggered NADPH oxidase-ROS-JNK pathway, we investigated whether Traf4 and Pyk2 are required for NADPH oxidase activation enhanced by Hic-5 using a dual transfection of p-Hic-5 coupled with siRNA of Traf4 or Pyk2. As shown in Supplementary Fig. 5A, transfection of p-Hic-5 coupled with control siRNA elevated the NADPH oxidase activity in HCC413 by 2.8-fold compared with the p-CMV/control siRNA group. Remarkably, depletion of Traf4 and Pyk2 decreased the Hic-5-enhanced NADPH oxidase activity by 36% and 34%, respectively, in HCC413 (Supplementary Fig. 5A). On the other hand, RT-PCR analysis demonstrated depletion of Traf4 or Pyk2 decreased expression of Hic-5 and Zeb-1 mRNA in HCC413 (Supplementary Fig. 5B). The efficiency of depleting Pyk2 by siRNA (by 90%) was demonstrated in the western blot of Pyk2 (Supplementary Fig. 5C). However, the efficiency of Traf4 knockdown was hard to be evaluated, due to the much lower sensitivity of Traf4 western blot using whole cell lysate (data not shown) than that of Traf4 Western using the Traf4-enriched Hic-5 immunopreciptates (see IP Hic-5/western Traf4 analysis in Fig. 3c and Supplementary Fig. 4)

### Hic-5 expression was positively regulated by NADPH oxidase-ROS-JNK pathway

**Fig. 4:**
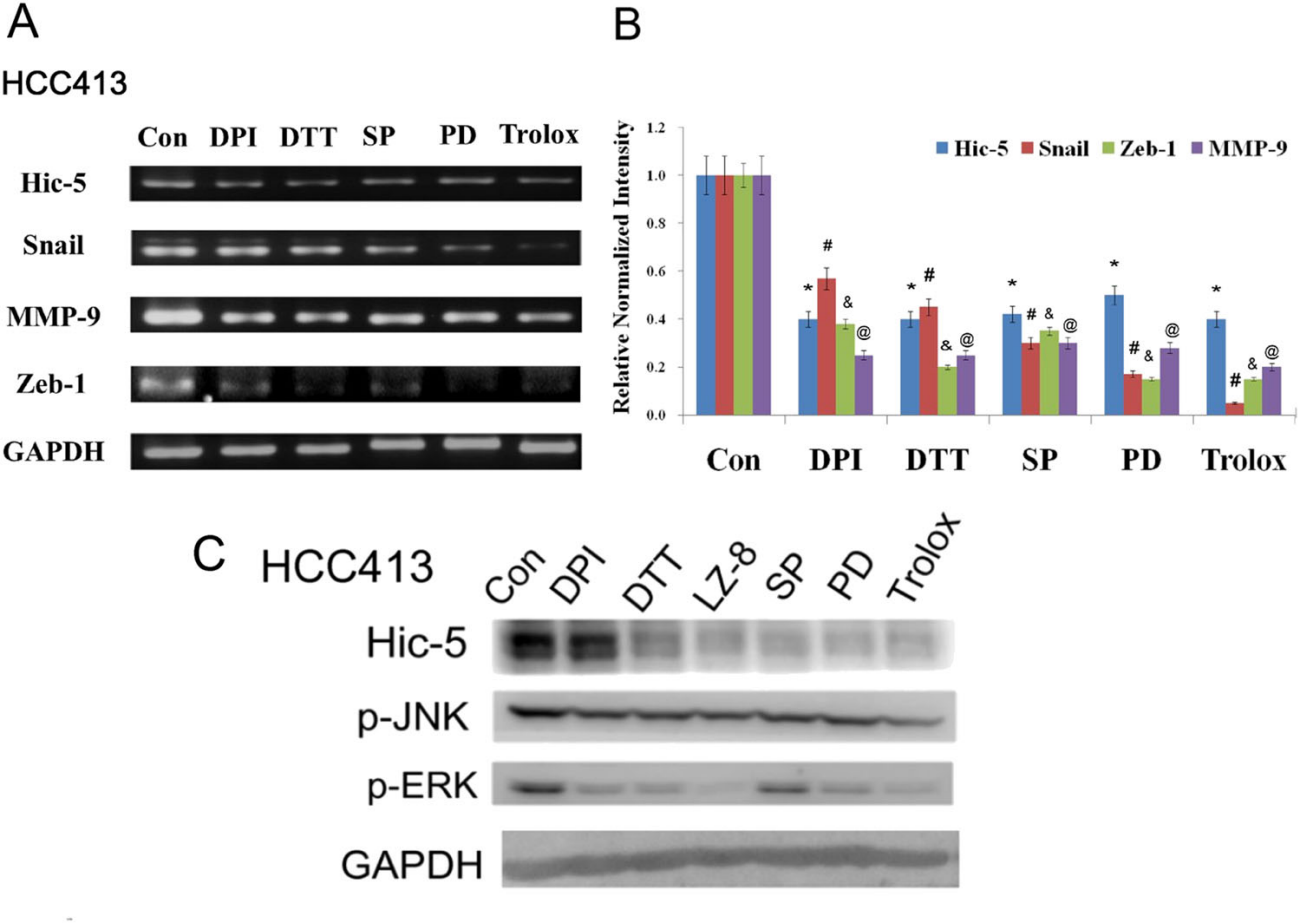
Inhibitors of ROS-JNK pathway prevented Hic-5 expression. HCC413 cells were untreated (con) or treated with indicated inhibitors. RT-PCR (**a**) and western blot (**c**) of indicated molecules were performed using GAPDH as an internal control. Panel (**b**) is the quantitative figures of (**a**). The data shown are averages from five reproducible experiments with coefficient variation (C.V.) of 8.0%. (*, #_,_ $_,_^@^) represent the statistically significant differences (*p* < 0.05, *N* = 5) between each of the indicated inhibitor-treated group and respective untreated (Con) group. Panel (**c**) is a representative of three reproducible results. DPI: Diphenyleneiodonium chloride, DTT: dithiothreitol, PD: PD98059, SP: SP610025

On the other hand, we investigated whether Hic-5 also locates downstream of NADPH oxidase-ROS-JNK cascade. In our previous report, Hic-5 can be suppressed by ROS scavengers such as dithiotheritol (DTT, a –SH containing antioxidant) and a JNK inhibitor such as SP600125 (SP) in HCC329^5^, we further examined whether more inhibitors of signal molecules within the NADPH oxidase-ROS-JNK cascade can also suppress Hic-5 expression in HCC413. For this purpose, in addition to DTT (0.3 mM) and SP (10 μM), inhibitor of NADPH oxidase diphenyleneiodonium chloride (DPI, 30 nM), an antioxidant Trolox (analog of vitamin E), an inhibitor of MEK (upstream of ERK) PD98059 (10 μM) were employed. Both mRNA (Fig. 4a, b) and protein (Fig. 4c, d) of Hic-5 coupled with phosphorylation of JNK and ERK (Fig. 4c, d) were suppressed by these inhibitors by 20–80% in HCC413. Similarly, mRNA of Snail, Zeb1 and MMP9 in HCC413 (Fig. 4a, b) and HGF-induced activation of NADPH oxidase in HCC340 (Supplementary Fig. 3D) were greatly suppressed by the aforementioned inhibitors by 30–80%. One exception is that PD98059 did not significantly prevent HGF-induced activation of NADPH oxidase (Supplementary Fig. 3D). Also, quantitative RT-PCR confirmed the suppression of mRNAs of Hic-5, Snail and MMP-9 by 10–95% in HCC413 treated with these inhibitors (Supplementary Fig. 2C). In addition, LZ-8 also effectively suppressed the level of Hic-5 protein and phosphorylation of JNK and ERK in HCC413 (Fig. 4c, d).

### Hic-5 transcription is positively regulated by ROS-JNK-c-jun pathway

**Fig. 5:**
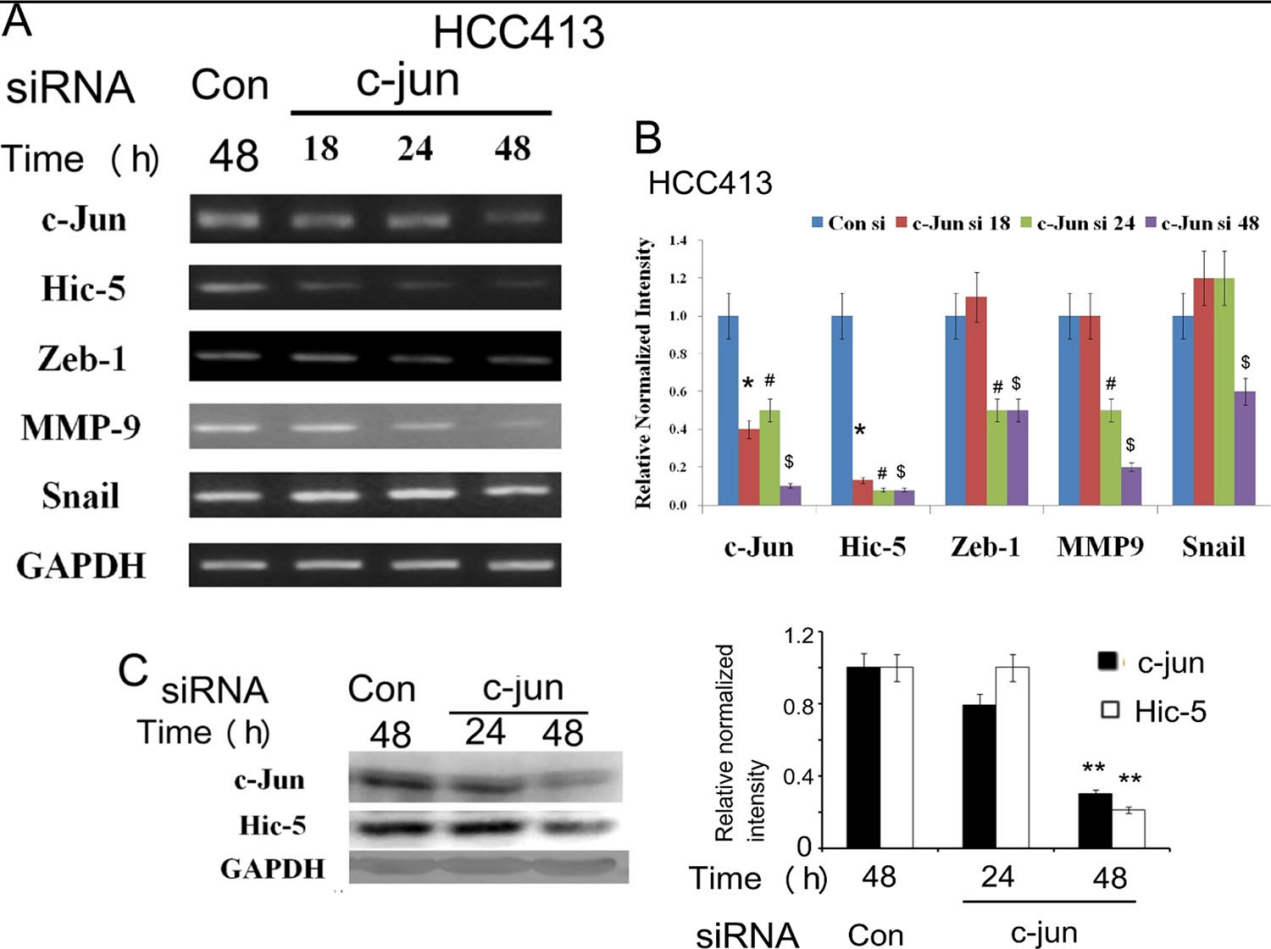
c-jun was required for gene expression of Hic-5 and mesenchymal markers. HCC413 cells were transfected with Control or c-jun siRNA for indicated time. RT/PCR (**a**) and western blot (**c**) of indicated molecules were performed. GAPDH was used as an internal control. Panel (**b**) is the quantitative figure of (**a**). Relative normalized intensity was calculated, taking the data of Con siRNA group as 1.0. The data shown are averages of three reproducible experiments with coefficient variation (C.V.) of 8.0–9.5%. (*, #_,_ $) represent the statistically significant differences (*p* < 0.05, *N* = 3) between each of the cells transfected with c-jun siRNA for the indicated times (18, 24, and 48 h) and that of the respective control siRNA (Con si) group at each time point. The quantitative figure of (**c**) is shown in the lower panel. The data shown are averages from three reproducible experiments with coefficient variation (C.V.) of 10.5 %. (**) represent the statistically significant difference (*p* < 0.005, *N* = 3) between the indicated group and the Con siRNA group for each molecule

**Fig. 6:**
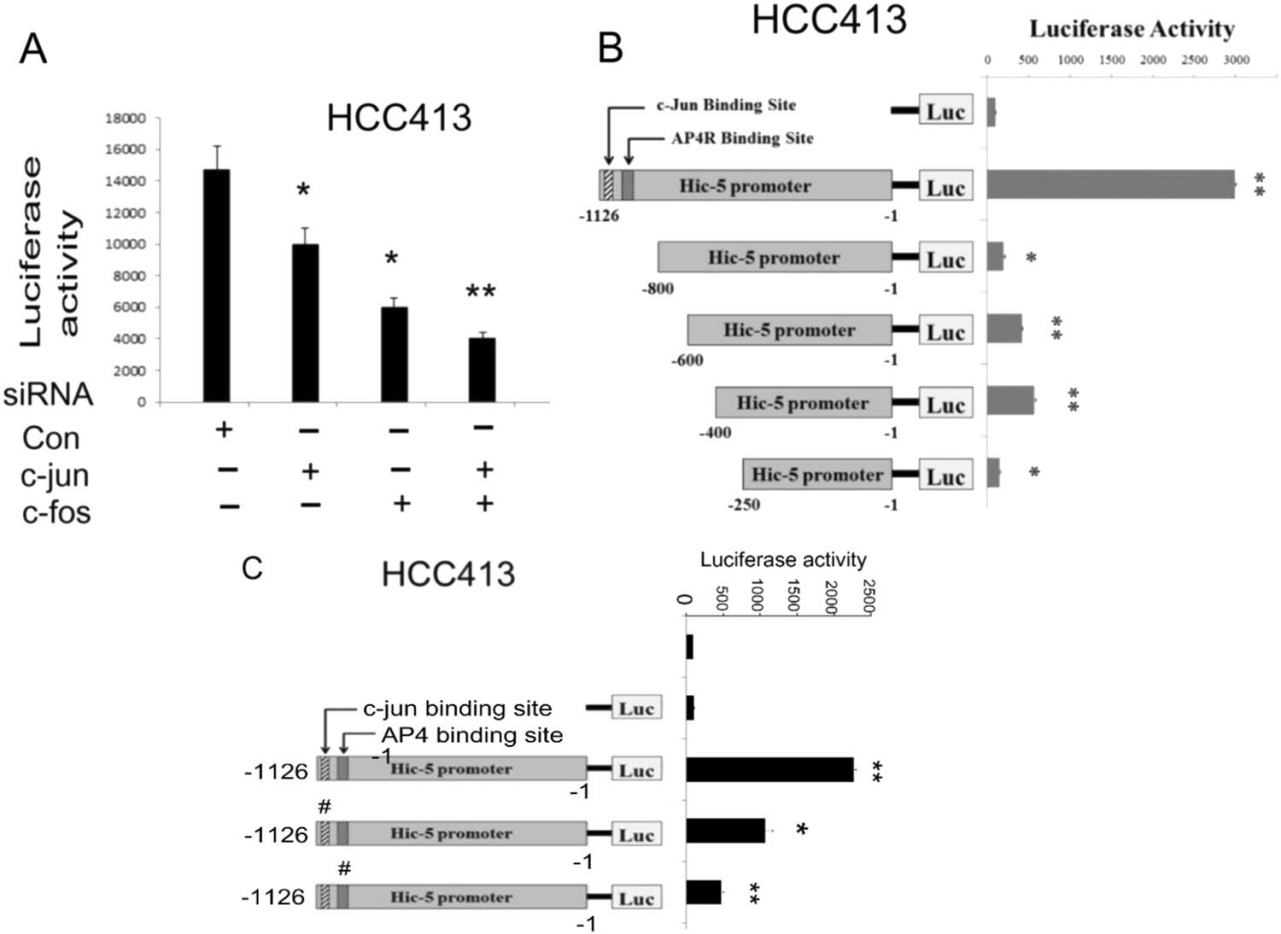
c-jun and c-fos protein and critical transcription factor binding regions were required for Hic-5 promoter activation. **a** HCC413 cells were transfected with control siRNA, c-jun, and/or c-fos siRNA for 24 h, followed by transfection of the full-length Hic-5 promoter proHic-5 (1126); **b** HCC413 cells were transfected with proHic-5 (1126) or various 5′ deleted promoters; **c** HCC413 were transfected with wild-type proHic-5 (1126) or various proHic-5 (1126) mutants with alterations in c-jun and AP4 putative binding region. Subsequently, single luciferase assay were performed, taking the data of pGL3 as 1.0. In (**c**), “#” highlights the promoter mutants with alteration at c-jun or AP4 binding regions as indicated. (**, *) represent the statistically significant difference (*p* < 0.005, *p* < 0.05, respectively, *N* = 4, C.V. 8.0–9.5%) of promoter activity between the indicated groups with control siRNA group (**a**) or between the indicated deletion promoter and the full-length Hic-5 promoter group (**b**) and (**c**)

We further examined whether Hic-5 expression is positively regulated on the transcriptional level downstream of ROS-JNK pathway using pGL3 based Hic-5 promoter constructs. Initially, a full-length Hic-5 promoter proHic-5(1126), containing 1126 bp upstream of translational initiation site, was employed. As shown in luciferase assays of proHic-5(1126), Hic-5 promoter was constitutively active in HCC413 (Supplementary Fig. 6A) and was induced by HGF in HCC340 (Supplementary Fig. 6B). Moreover, DPI, DTT and SP100625 suppressed both the constitutive (Supplementary Fig. 6A) and HGF-induced (Supplementary Fig. 6B) Hic-5 promoter activity by 40–80%. These strongly indicated that transcriptional activation of Hic-5 was positively regulated by NADPH oxidase-ROS-JNK pathway. Since c-jun is the downstream transcriptional factor of ROS-JNK signaling, we examined whether it was critical for Hic-5 expression. As demonstrated in Fig. 5, knockdown of c-jun not only decreased Hic-5 expression at both mRNA (Fig. 5a, b) and protein (Fig. 5c) level by 88% compared with that in control group (*p* = 0.005, *N* = 5) but also greatly decreased the amount of Snail, Zeb1, and MMP9 mRNA in HCC413 (Fig. 5a, b). Quantitative RT-PCR further confirmed the decrease of mRNAs of Hic-5, Snail and MMP9 by 92%, 80%, and 75%, respectively, in HCC413 transfected with c-jun siRNA for 48 h compared with those in the cell transfected with control siRNA (Supplementary Fig. 2D). Moreover, depletion of c-jun or c-fos (which is the transcription factor associated with c-jun as the heterodimer AP-1) also significantly attenuated the promoter activity of Hic-5 (Fig. 6a). Interestingly, depletion of both c-jun and c-fos decreased the Hic-5 promoter activity more than depletion of either transcriptional factor alone (Fig. 6a). Together, these results suggested that Hic-5 promoter was positively regulated by c-jun and/or AP-1 transcriptional system.

### Deletion mapping and site directed mutagenesis for identifying ROS-JNK responsive element on Hic-5 promoter

We further investigated the critical transcription factor binding regions on Hic-5 promoter responsible for activation of Hic-5 gene. According to Genomatix software, there is one putative binding motif of c-jun A**TGG**TCA locating at 1109–1116 bp upstream of the translational initiation site (−1116 to −1109 bp). In addition, an AP4 region TGCCCAGAGCTGCCTCC located at −958 to −941 bp, nearby the c-jun region. Since AP-4 was known to be critical for EMT and progression of a lot of tumors^24^, the involvement of AP4 binding region was also examined. For this purpose, deletion constructs proHic-5(800), proHic-5(600), proHic-5(400), proHic-5(250) which excluding 300, 500, 700, and 850, respectively, bp from 5′ end of full-length promoter proHic-5(1126) were employed. As demonstrated in Fig. 6b, activities of proHic-5(800) and all the other deletion constructs decreased to below 10% of full-length promoter, indicating the promoter regions responsible for Hic-5 transcription locate on the region between −1126 and −800 bp, upstream of translational initiation site. Since this promoter fragment contained both c-jun and AP4 region, they are very probable required for Hic-5 promoter activation. To confirm this issue, site directed mutagenesis on the putative c-jun (TGG → AAA) and AP4 (GCT → AAA) binding region within full-length Hic-5 promoter, proHic-5(1126), was performed. As demonstrated in Fig. 6c, activities of both site directed mutant constructs decreased to below 10% of wild-type proHic-5(1126). Together, these results indicated that the putative c-jun and AP4-binding region were required for Hic-5 promoter activation.

## Discussion

**Fig. 7:**
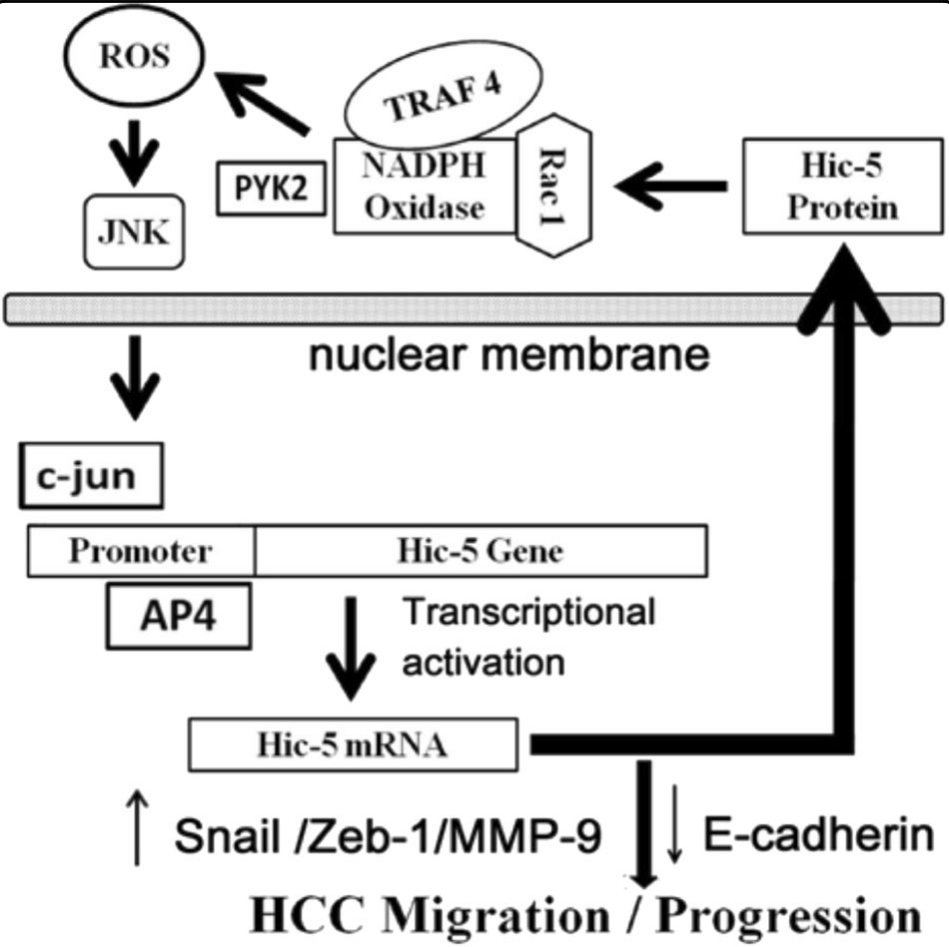
Scheme for Hic-5 mediated positive feedback NADPH oxidase-ROS-JNK-c-jun cascade, regulating EMT markers. According to the data shown in this report, Hic-5 can be induced by ROS-JNK pathway and transactivated by c-jun and AP4 which may in turn interact with Rac1 and Traf4, and Pyk2 triggering activation of NADPH oxidase, ROS generation and JNK phosphorylation thus sustaining the signal transduction. In this way, Hic-5 may play a central role in mediating the positive feedback ROS-JNK signaling circuit which upregulates Snail, Zeb-1, and MMP-9 and downregulate E-cadherin for triggering HCC migration and progression of HCC

In this paper, we found Hic-5 plays as a central mediator for sustaining ROS signaling in a positive feedback manner. The phenomenon that Hic-5 cross reacts with ROS-JNK signal cascade has been mentioned in our previous report using HCC329^5^. Herein, a lot of missing links in the Hic-5-ROS-JNK signal circuit were clarified and the EMT related genes regulated by Hic-5 for HCC progression were identified. On the upstream, we found Hic-5 interact with the regulators of NADPH oxidase, including Rac-1, Traf4, and pyK2, to activate its enzyme activity required for ROS generation. In the downstream, we confirm that ROS-JNK pathway indeed is responsible for upregulation of Hic-5 gene expression via the transcriptional factor c-jun coupled with AP4. The Hic-5 thus induced in turn reactivate ROS and JNK, establishing a positive feedback signal circuit that regulates the expressions of EMT related genes, including E-cadhedrin, Snail, MMP9, and Zeb-1, required for HCC migration and progression^5^ (see Scheme in Fig. 7).

Previous studies have found the bi-directional interaction of Hic-5 with other major signaling pathways. Whereas TGFβ was known to be an inducer of Hic-5^7,25^, TGFβ-induced signaling can also be positively regulated by Hic-5. Hic-5 may promote TGFβ-induced signaling by binding to and inactivating the inhibitory Smads, Smad3^26^, and Smad7^27^ leading to enhanced TGF-β/Smad2 signaling required for EMT. In addition, while activation of ERK was required for Hic-5 to promote endothelial cell migration^28^, Hic-5 may serve as a scaffold protein that specifically activates the MAPK cascade^29^.

In similar with the aforementioned signaling properties of Hic-5, ROS was also well known to be capable of mediating sustained signaling via cross talk^30–33^. A positive feedback loop was found between ROS and MLK3/Braf/ERK cascade for invasion of colorectal cancer cells. Also, IL-6^31,34^ and TGF-β^35^ may cross talk with ROS to trigger progression of prostate tumor and lung cancer, respectively. On the transcriptional level, ROS can activate Snail which in turn induced intracellular ROS production, thus creating a self-regulating loop which leads to EMT^36,37^.

Previously, the role of Hic-5 in ROS generation during endothelial cell migration has been suggested^11^. In this context, Hic-5 may serve as an adaptor for association with TRAF4 and p47^*phox*^ which initiate Rho GTPase activation required for NADPH oxidase-dependent ROS production^11^. Consistently, Hic-5 was among the TRAF4/p47^phox^/Hic5/Pyk2 complex associated with the platelet collagen receptor, GPVI, a major platelet collagen receptor required for ROS formation^20^. The role of Pyk2 in this pathway is also intriguing. It has been reported that Tyr-881 on Pyk2 became highly phosphorylated during EMT and migration of murine mammary NMuMG epithelial cells^23^. Also, Pyk2 was responsible for RhoC-triggered MAPK signaling for prostate cancer progression^38^. In our results, Hic-5 may associate with Traf4 and Pyk2 in HCC413 (Supplementary Fig. 4). Also, increased association of Hic-5 with Traf4 and Rac-1 (Fig. 3c), Rac-1 activity (Fig. 3a) and phosphorylation of Pyk2(Tyr881) (Fig. 2b) were observed in HCC340 overexpressing Hic-5. Importantly, Traf4 and Pyk2 not only could mediate Hic-5 triggered NADPH oxidase activation (Supplementary Fig. 5A), but also were essential for expression of Hic-5 and the downstream EMT transcriptional marker Zeb-1 (Supplementary Fig. 5B), implicating they act both upstream and downstream of Hic-5. Collectively, our results suggest that Hic-5 may associate with Rac-1/Traf4/Pyk2 to activate NADPH oxidase-dependent ROS generation required for activation of downstream JNK signaling. It is tempting to observe whether depletion of the aforementioned Hic-5-interacting molecules will suppress ROS-JNK cascade and HCC progression.

We have shown the constitutive and HGF-induced Hic-5 expression in this and previous study^5^. In addition to the induction by HGF, Hic-5 expression can also be induced during TGFβ1-triggered senescence of osteoblastic cell line^4^, angiotensin II-induced abdominal aortic aneurysm (AAA) development^29^, methylmercury-induced ER stress^39^, and *Escherichia coli*-induced prostatic inflammation^40^. In spite of these, the detailed transcriptional mechanisms for Hic-5 gene expression induced via different signaling pathway have not been delineated thus far. Previous study indicated Hic-5 promoter contains an evolutionarily conserved CArG element which is indispensable for myocardin-induced transactivation of Hic-5. Also, they found that serum responsive factor (SRF) binds to this CArG element and is essential for TGF-β-mediated induction of Hic-5^41^. In this study, we found c-jun, the downstream effector of JNK cascade are required for the induction of Hic-5 transcription. Promoter assay also indicated the requirement of the putative c-jun and AP4 binding motif essential for Hic-5 promoter activation. In the future, whether c-jun and AP4 directly bind on the indicated region can be investigated by ChIP assay and EMSA. In addition, we found both c-jun and c-fos (the transcription factor associated with c-jun as heterodimer, AP-1) were required for the promoter activity of Hic-5 (Fig. 6a). Specifically, depletion of both c-jun and c-fos decreased the Hic-5 promoter activity more than that of either transcriptional factor alone (Fig. 6a). This suggested that c-jun/c-fos (AP-1) was also involved in Hic-5 promoter activation. However, we did not find typical AP-1 binding region on Hic-5 promoter according to Genomatix software. It is possible that there is an un-identified AP-1 region within the Hic-5 promoter or, alternatively, c-fos is required for c-jun to bind on its putative c-jun region as identified in this study (Fig. 6b, c), an issue worthy of further investigation.

ROS signaling was known to be essential for EMT that facilitates tumor progression. A lot of mesenchymal markers including transcription factors Snail, Twist, and Zeb1 were known to be responsible for regulating EMT markers such as E-cadherin and MMP-9 for HCC progression. In our results, Hic-5 (Fig. 1d, e) and c-jun (Fig. 5a) were required for expression of a lot these mesenchymal markers, including Snail, Zeb1, and MMP9. On the other hand, overexpression of Hic-5 resulted in enhancement of the aforementioned mesenchymal markers (Fig. 2b, c) and decrease of E-cadherin (Fig. 2c). These results not only suggested the essential EMT markers were the effector genes downstream of Hic-5-ROS-JNK-c-jun cascade but also indicated that c-jun and AP-4 transcriptional system are involved in the regulation of these genes, which is worthy of investigation.

In conclusion, Hic-5 play central role in positive feedback Rac-1-Traf4-NADPH oxidase-ROS-JNK-c-jun signal cascade for triggering HCC migration, invasion, and progression^5^. Due to the limited expression of Hic-5 in normal tissue^42^, it can be a promising therapeutic target for preventing HCC metastasis.

## Materials and methods

### Cell lines, plasmid and chemicals

HCC413 and HCC340, the patient-derived cell lines, were established on January 2014 from parts of HCC tissues obtained from surgery according to the methodology described in our previous report^45^. Briefly, HCC tissues were pretreated with collagenase and cultivated on the mitomycin C-treated NIH3T3 feeder layer for 4–6 passages to select the HCC cell lines. Cell lines obtained from this method were authenticated by STR, analyzing the characteristic of microsatellite patterns of patient’s genomes (unpublished results). Homogenous HCC cell populations were obtained and the sustained proliferation ability (over 20 passages) and metastatic potentials were tested in vitro and in vivo after more than 40 passages. The characteristics of the HCC tumor cell lines were validated by detecting HCC tumor makers such as Glypican 3 (GCP3)^46^. HGF was from Peprotech (Rocky Hill, NJ, USA). Dithiotheritol was from Sigma (Milwaukee, DC, USA). Antibodies for Hic-5, phosphorylated JNK/ERK, c-jun/phosphorylated c-jun, and GAPDH were obtained from Santa Cruz Biotechnology, Inc. (California, CA, USA). Hic-5 c-DNA expression plasmid (TGFB 1I1) was from OriGene Technologies, Inc. (Washington, DC, USA).

### Transwell migration assay

Cells were seeded on a 24-well transwell migration insert (Nalge Nunc International, Rochester, NY, USA) in a complete medium for 24 h. After appropriate treatments, cells that had migrated to the underside of the insert membrane were stained with 0.3% crystal violet. The cells on the topside of the insert membrane were rubbed with a cotton swab. The migrated cells on the underside were imaged using phase contrast microscopy with ×200 magnification. Quantitation of the migrated cell was performed by measuring the intensity of crystal violet staining, using ImageJ software.

### Invasion assay

Cells were seeded on a 24-well transwell migration insert (Nalge Nunc International, Rochester, NY, USA) coated with Matrigel (Corning, MA, USA) in a complete medium for 24 h. After appropriate treatments, cells that had invaded to the underside of the insert membrane were stained with 0.3% crystal violet.

### Conventional RT-PCR and real-time RT-PCR

Conventional RT-PCR was performed according to that described in our previous report^44^. Quantitative real-time qRT-PCR was performed using EZtime Real-Time PCR Premix (Yeastern Biotech Co, Taipei, Taiwan) with gene-specific primers and probes on a sequence-detection system (ABI 7900HT Fast Real-Time PCR System, Applied Biosystems, Foster City, CA).

### Western blot

A western blots were performed according to our previous studies^5^. The band intensities on the blots were quantified using ImageJ software.

### Cytometric analysis for ROS generation

Cytometric analysis for ROS generation was performed as described in our previous report^5^. Briefly, intracellular H_2_O_2_ and superoxide anions O_2_^−^ production were monitored by oxidation of permeable dye 2,7-dichlorodihydrofluorescein diacetate (DCF-DA) and Dihydroethidium (DHE), respectively, which react with ROS to form the fluorescent product. The cells were incubated with 20 μmol/L DCFDA or DHE for 30 min and washed twice with PBS. After required treatment, the cells were then harvested for flow cytometry as described previously^5^. Each determination is based on the mean fluorescence intensity (MFI) of 5000 cells.

### Rac-1 assay

Rac-1 assay was performed according to our recent report^43^.

### NADPH oxidase assay

NADPH oxidase activity was measured as described previously. Briefly, the cells were sonicated in 3 × 10-s bursts at 30% power. The cell homogenate was added to the wells of a bioluminescence plate followed by measurement of NADPH oxidase activity in a 50 mM phosphate buffer, containing 1 mM EGTA, 150 mM sucrose, and 10 μM lucigenin. O_2_^−^ production will be stimulated with NADPH (200 μM), succinate (5 mM) (with ANM), or xanthine (100 μM). The NADPH-dependent O_2_^−^ production was determined from the ratio of mean chemiluminescent light units to total protein level.

### RNA interference of Hic-5 and overexpression of Hic-5

Hic-5 expression was transiently knocked down by 25 nM Hic-5 siRNA (Thermo Scientific, Dharmacon, USA) for 48 h, according to the manufacture’s protocol. For overexpression of Hic-5 in HCCs, the cells were transfected with Hic-5 cDNA expression plasmid (TGFB 1I1) driven by CMV promoter (p-Hic-5) for 48 h. Double transfection of p-Hic-5 coupled with Traf4 or Pyk2 siRNA was performed using DharmaFECT Duo Transfection Reagent (Thermo Scientific, Dharmacon, USA). The elevation or depletion of Hic-5 was validated by Western blot and RT-PCR.

### Constructions of various promoter plasmids for deletion mapping

The promoter regions in the full-length promoter plasmids ProHic-5(1126) were amplified from genomic sequence of Hic-5 encompassing 1126 bp upstream of translation start site. The PCR products were ligated into pGL3 vector (Promega, Madison, WI, USA). The promoter plasmids proHic-5(800), proHic-5(600), proHic-5(400), proHic-5(250) were derived from ProHic-5(1126) by double digestion with various restriction enzymes followed by filling in the restriction site overhangs by Klenow enzyme. Subsequently, the digested DNA fragments were ligated into pGL3 vector.

### Construction of site directed mutants of full-length Hic-5 promoter

To obtain the site directed mutants of Hic-5 promoter, the core sequence matrix on the indicated transcriptional factor binding regions within wild type Hic-5 promoter proHic-5(1126) were changed into random sequence by KOD-plus-mutagenesis kit according to manufacturer’s protocol (TOYOBO, Japan).

### Promoter assay

Promoter assay was performed according to our previous report^44^. Briefly, transfections of the cells with full-length promoter and various truncated formats of Hic-5 promoter inserted in the pGL3 vector were performed using lipofetamin 2000. After required treatments, the cells were harvested and assayed for luciferase activity as described in manual protocol provided by the manufacture (Promega, Madison, WI, USA). The data were normalized with the cell numbers seeded on 24-well plate for luciferase activity assay.

### Statistical analysis

One-way analysis of variance using Student’s *t* test was conducted to evaluate the intensity differences between samples on the Western blot and RT/PCR and the differences in promoter activity between the indicated samples. Quantitative data were expressed as mean ± coefficient variation (CV), indicated by the error bars in each figure.

## Supplementary information

Supplemental Fig 1

Supplemental Fig 2

Supplemental Fig 3

Supplemental Fig 4

Supplemental Fig 5

Supplemental Fig 6
