# Supplementary material for: Hydrogen peroxide inducible clone-5 sustains NADPH oxidase-dependent reactive oxygen species-c-jun N-terminal kinase signaling in hepatocellular carcinoma
Source: Oncogenesis. 2019 Aug 6;8(8):40. doi: 10.1038/s41389-019-0149-8 (PMC6684519; doi:10.1038/s41389-019-0149-8)
Supplement: Supplementary file 1 — Supplemental Fig 1 [file 41389_2019_149_MOESM1_ESM.docx]

**Supplemental Fig. 1 Hic-5 is essential for invasion of HCC**


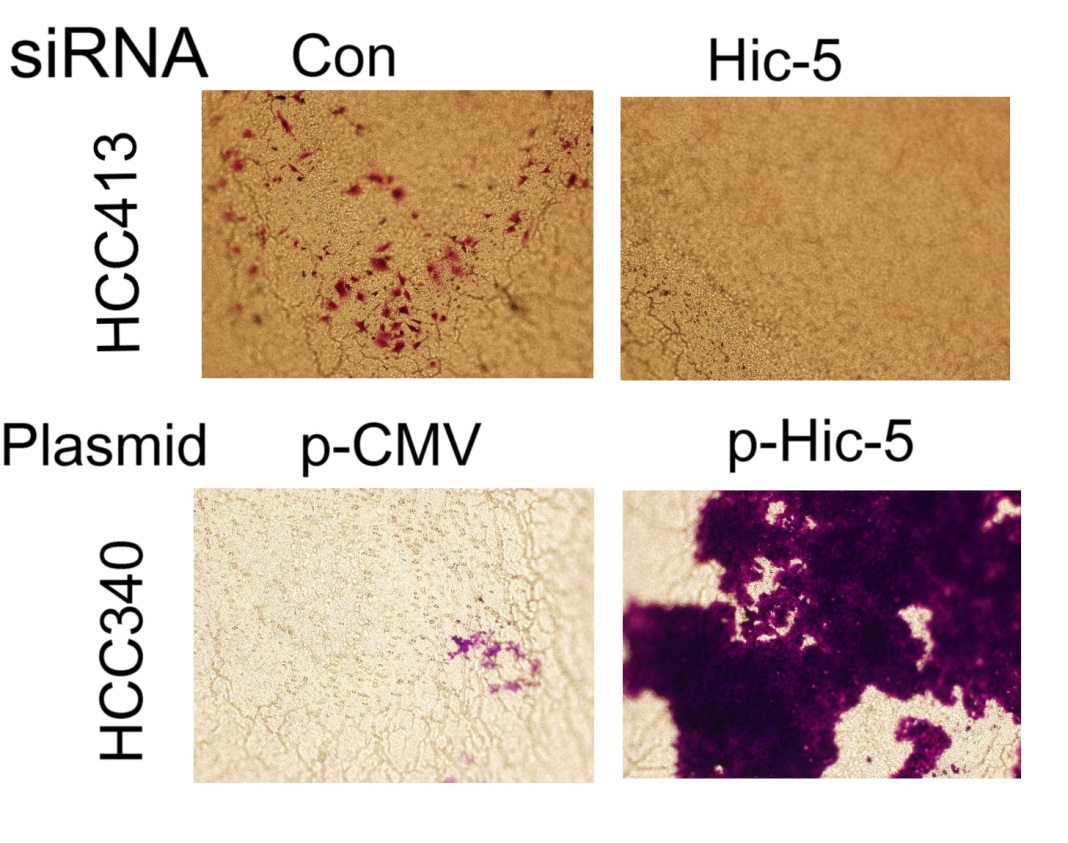


HCC413 cells were transfected with control siRNA or Hic-5 siRNA for 48 h (upper panel); HCC340 cells were transfected with p-CMV control vector or Hic-5 overexpression plasmid (p-Hic5) for 48 h (lower panel). Transwell matrigel invasion assay were perforemed. The data shown was a representative of two reproducible experiments.
