# Supplementary material for: Hydrogen peroxide inducible clone-5 sustains NADPH oxidase-dependent reactive oxygen species-c-jun N-terminal kinase signaling in hepatocellular carcinoma
Source: Oncogenesis. 2019 Aug 6;8(8):40. doi: 10.1038/s41389-019-0149-8 (PMC6684519; doi:10.1038/s41389-019-0149-8)
Supplement: Supplementary file 2 — Supplemental Fig 2 [file 41389_2019_149_MOESM2_ESM.docx]

Supplemental Fig. 2 Quantitative RT-PCR validating the regulation of gene expression of Snail and MMP-9 by Hic-5 and Hic-5 expression by c-jun

1. HCC413


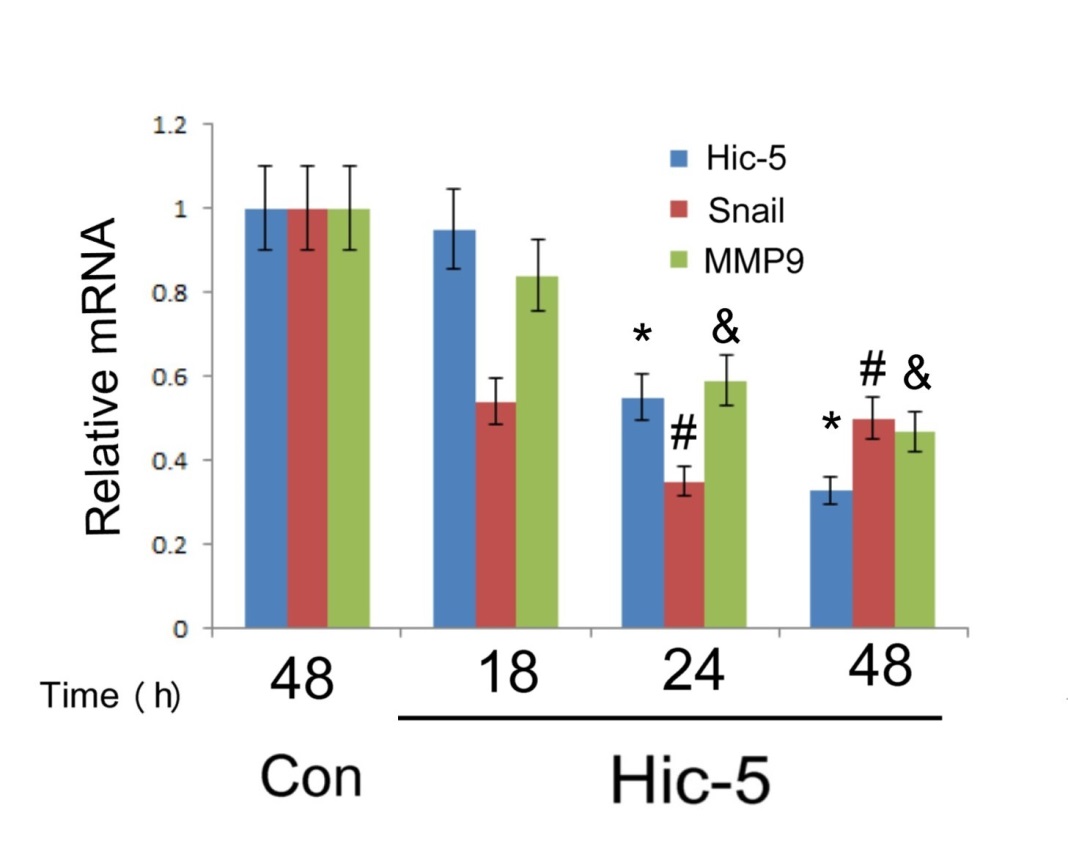


1. HCC340


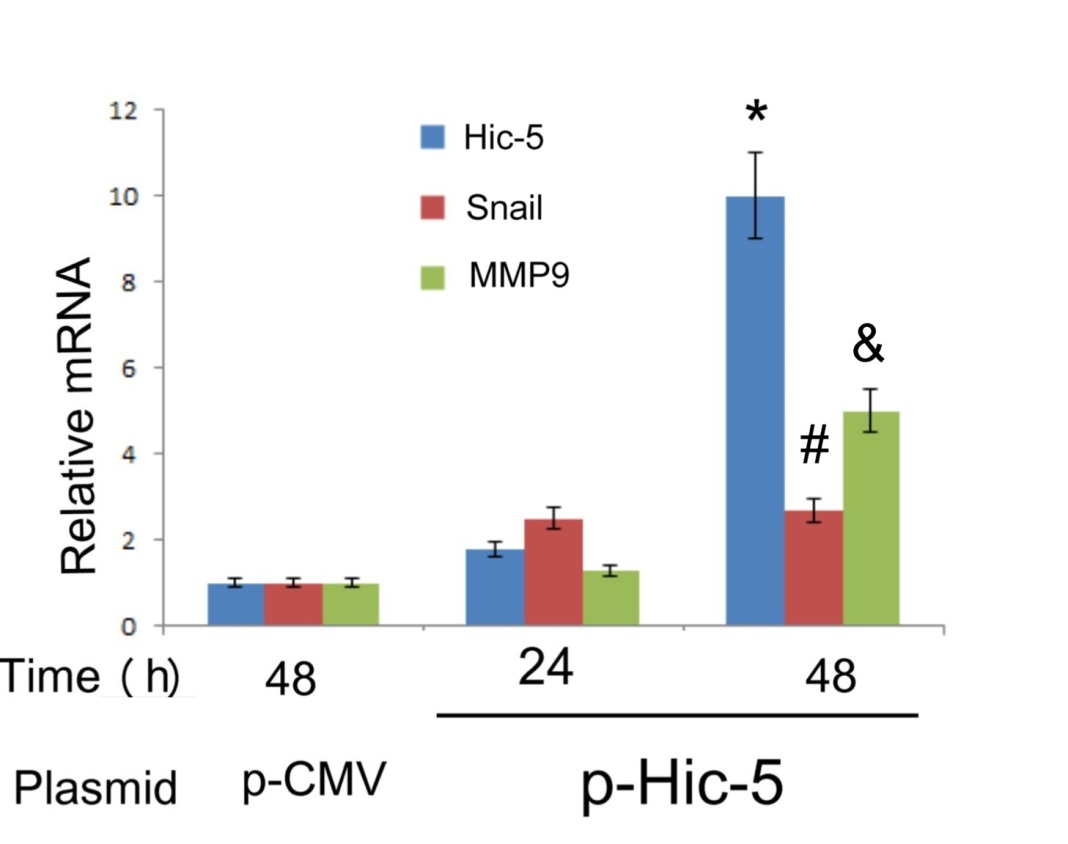


1. HCC413


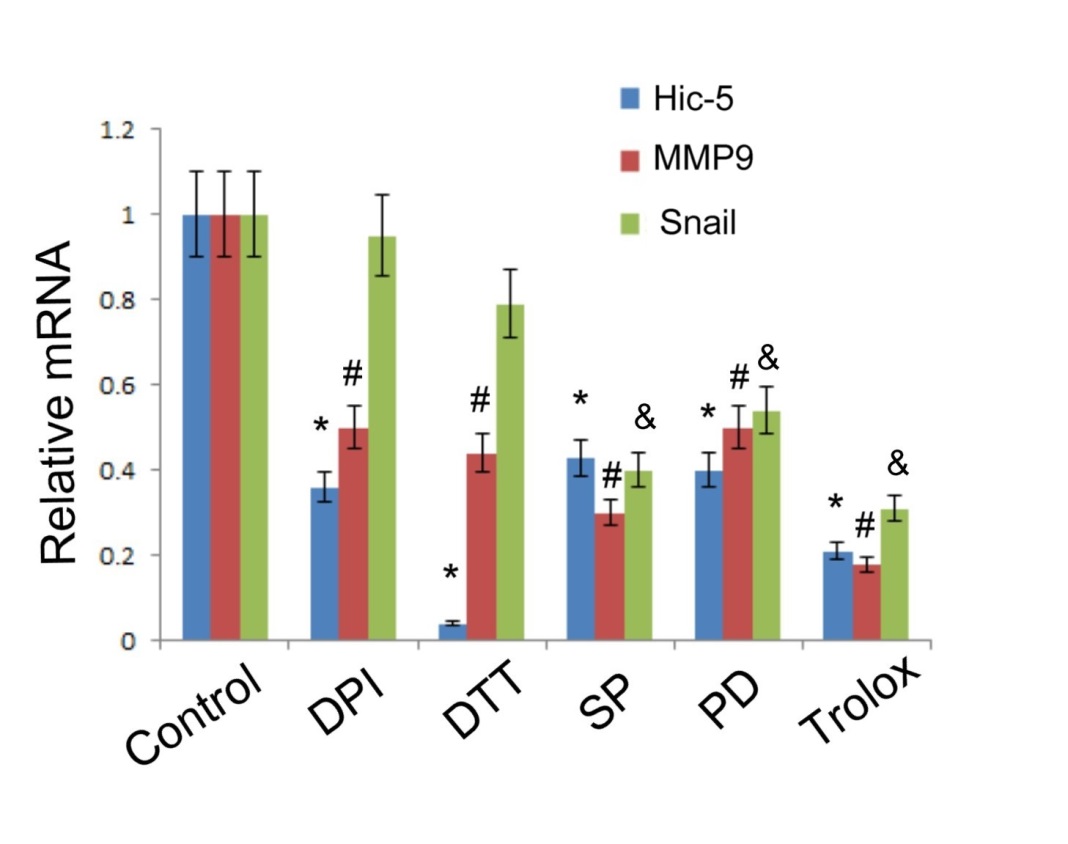


1. HCC413


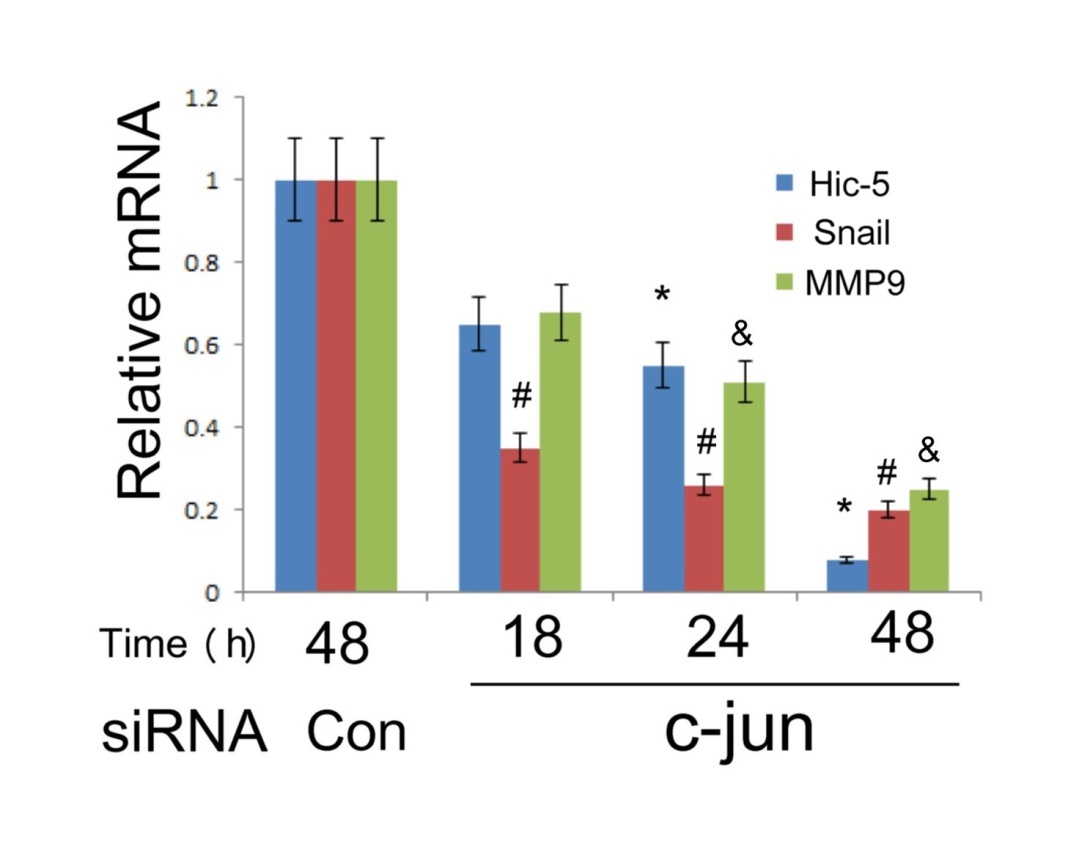


HCC413 were transfected with Control (Con) or Hic-5 siRNA for indicated time (A); HCC340 were transfected with pCMV vector or Hic-5 expression plasmid (p-Hic-5) for indicated times (B); HCC413 were untreated (Con) or treated with various inhibitors for 24 h (C); HCC413 were untransfected (non) or transfected with Control (Con) or c-jun siRNA for indicated time (D). Real time RT-PCRs of indicated molecules were performed. Relative mRNAs were calculated taking Control siRNA (A)(D), untreated (Con) (C) or p-CMV (D) group as 1.0. (*, #_,_ &) represent the statistically significant differences (p<0.05, N=3) between the indicated molecules in each of the indicated sample and that in the Control siRNA (A) (D), untreated (Con) (C) or p-CMV group (B). In (C) DPI: Diphenyleneiodonium chloride, DTT: dithiothreitol, PD: PD98059, SP: SP610025.
