# Supplementary material for: Hydrogen peroxide inducible clone-5 sustains NADPH oxidase-dependent reactive oxygen species-c-jun N-terminal kinase signaling in hepatocellular carcinoma
Source: Oncogenesis. 2019 Aug 6;8(8):40. doi: 10.1038/s41389-019-0149-8 (PMC6684519; doi:10.1038/s41389-019-0149-8)
Supplement: Supplementary file 3 — Supplemental Fig 3 [file 41389_2019_149_MOESM3_ESM.docx]

**Supplemental Fig. 3 Hic-5 was essential for constitutive and HGF-induced NADPH oxidase activity.**

**A B**


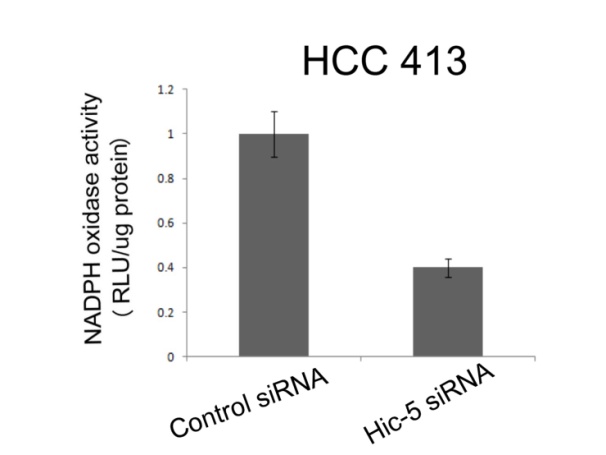

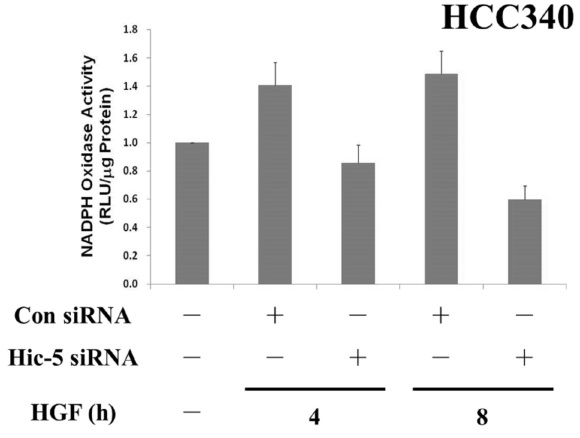


**C D**


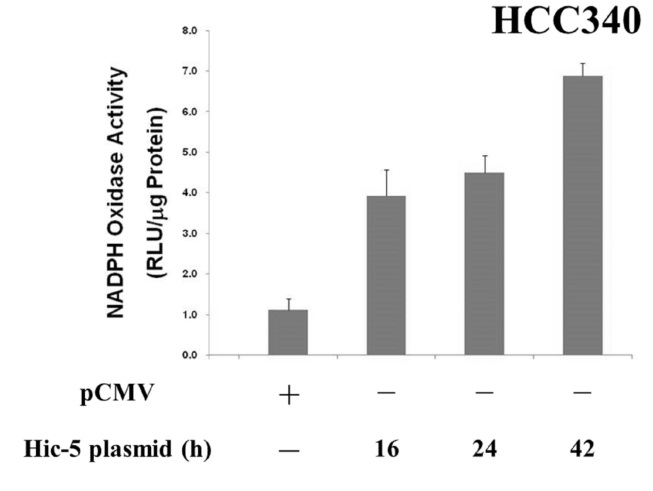

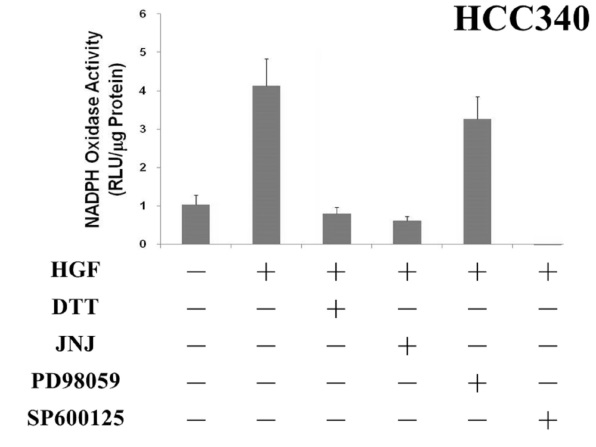


HCC413 (A) and HCC340 (B) were transfected with control or Hic-5 siRNA for 48 h followed by un-treatment (A) or treatment with HGF for 4 and 8 h (B); HCC340 were transfected with control (p-CMV) plasmid or Hic-5 expression plasmid for indicated times (C); HCC340 cells were untreated, treated with HGF or HGF coupled with indicated inhibitors for 4 h (D); NADPH oxidase activity assays were performed. Relative NADPH oxidase activities were calculated, taking control siRNA (A, B), p-CMV (C), untreated (D) as 1.0. The data shown are average from 2 reproducible experiments.
