# Supplementary material for: Hydrogen peroxide inducible clone-5 sustains NADPH oxidase-dependent reactive oxygen species-c-jun N-terminal kinase signaling in hepatocellular carcinoma
Source: Oncogenesis. 2019 Aug 6;8(8):40. doi: 10.1038/s41389-019-0149-8 (PMC6684519; doi:10.1038/s41389-019-0149-8)
Supplement: Supplementary file 4 — Supplemental Fig 4 [file 41389_2019_149_MOESM4_ESM.docx]

**Supplemental Fig. 4 Hic-5 interact with regulators of NADPH oxidase which can be suppressed by LZ-8**

**A**


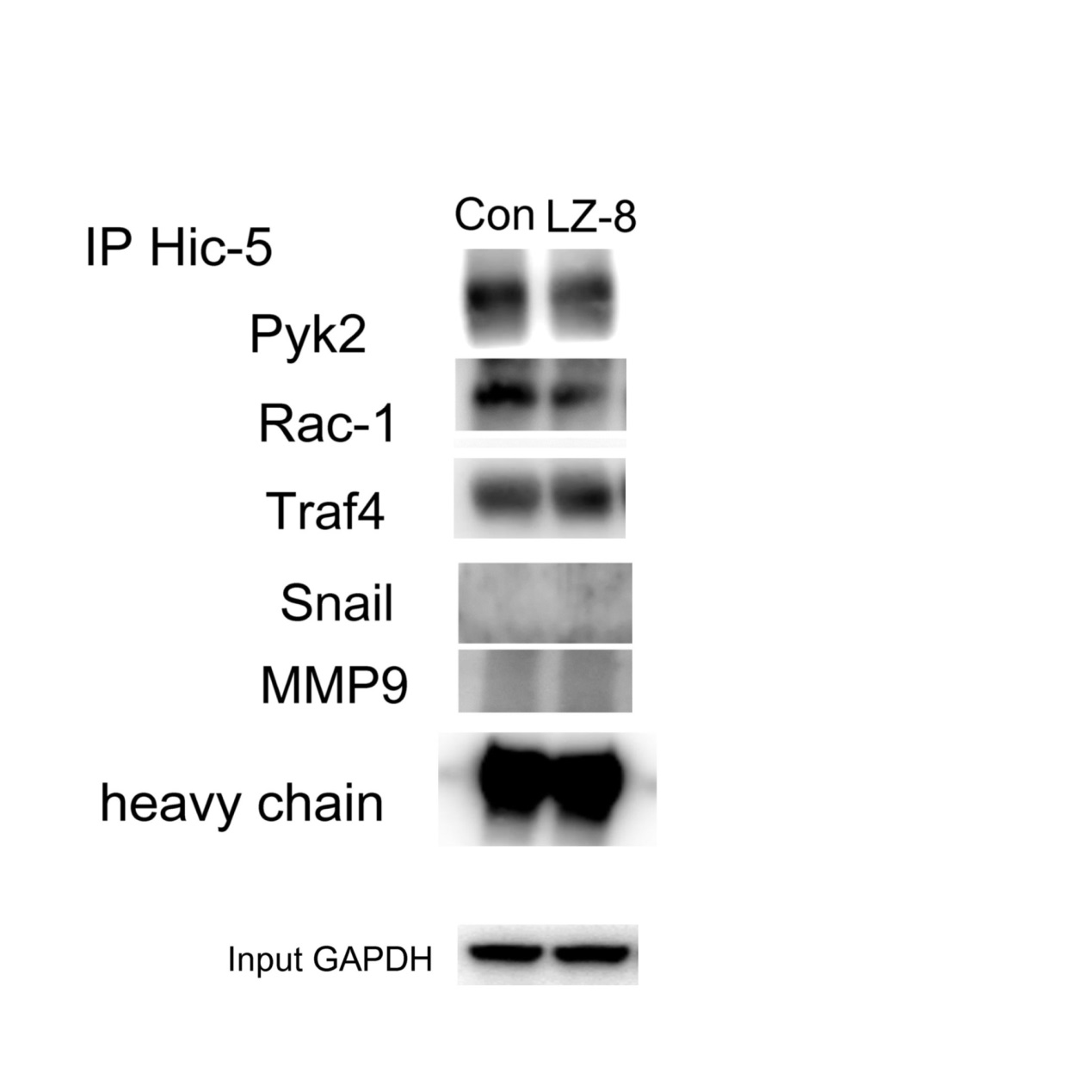


**B**


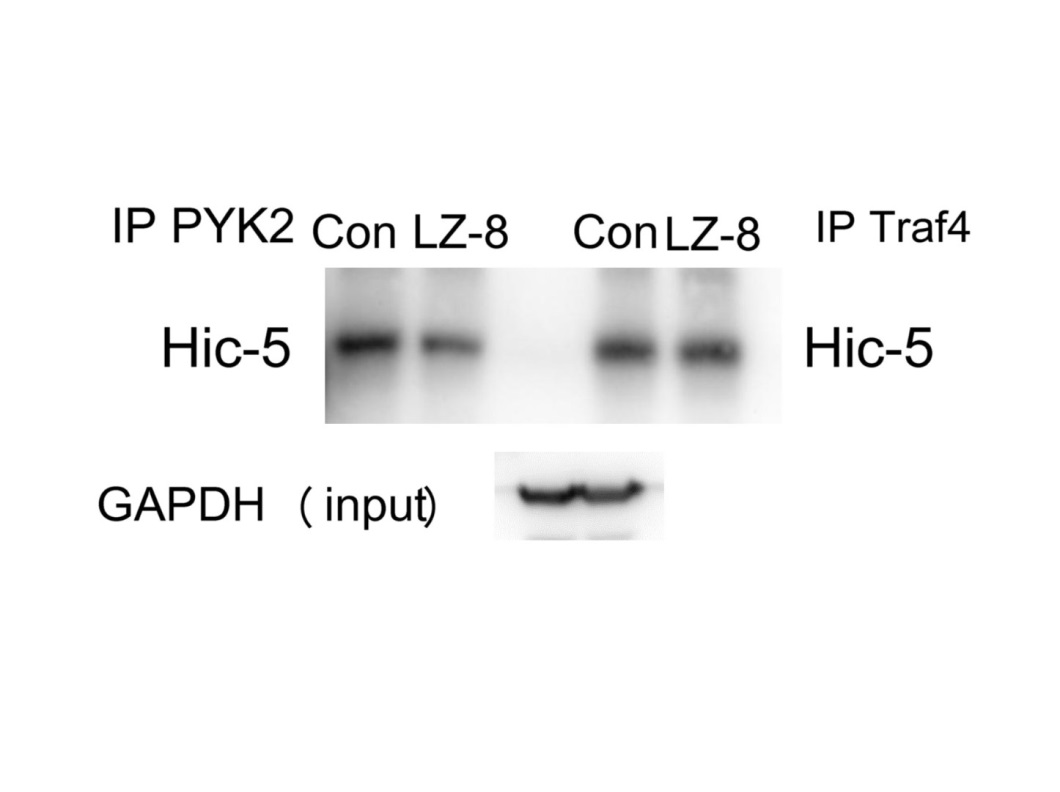


HCC413 cells were untreated (Con) or treated with LZ-8 (5 μg/ml) for 24 h, immunoprecipitation (IP) of Hic-5 followed by Western blot of the indicated molecules (A) or IP of indicated molecules followed by Western blot of Hic-5 (B) were performed. Western blot of GAPDH was included as an input. The data were representative of three reproducible experiments.
