# Supplementary material for: Hydrogen peroxide inducible clone-5 sustains NADPH oxidase-dependent reactive oxygen species-c-jun N-terminal kinase signaling in hepatocellular carcinoma
Source: Oncogenesis. 2019 Aug 6;8(8):40. doi: 10.1038/s41389-019-0149-8 (PMC6684519; doi:10.1038/s41389-019-0149-8)
Supplement: Supplementary file 5 — Supplemental Fig 5 [file 41389_2019_149_MOESM5_ESM.docx]

**Supplemental Fig. 5 Traf4 and Pyk2 mediated Hic-5 triggered NADPH oxidase activation and required for Hic-5 and Zeb-1 expression**

**A.**


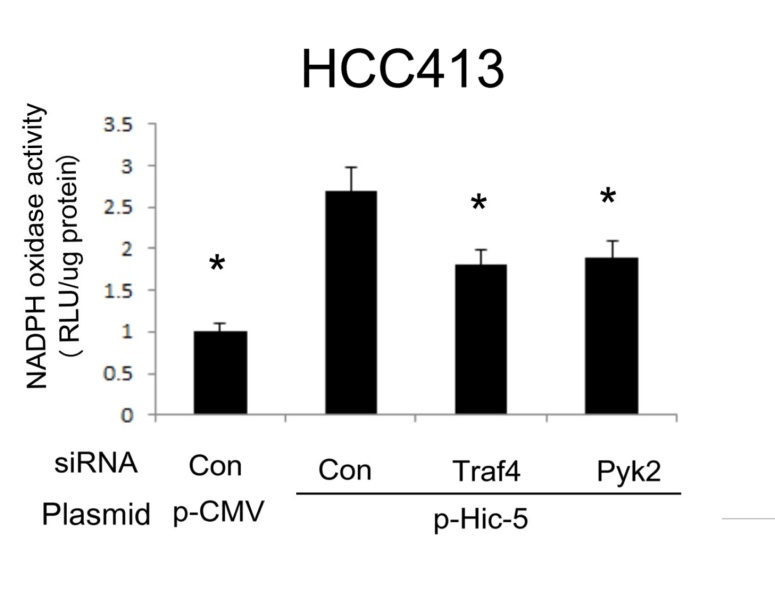


**B.**


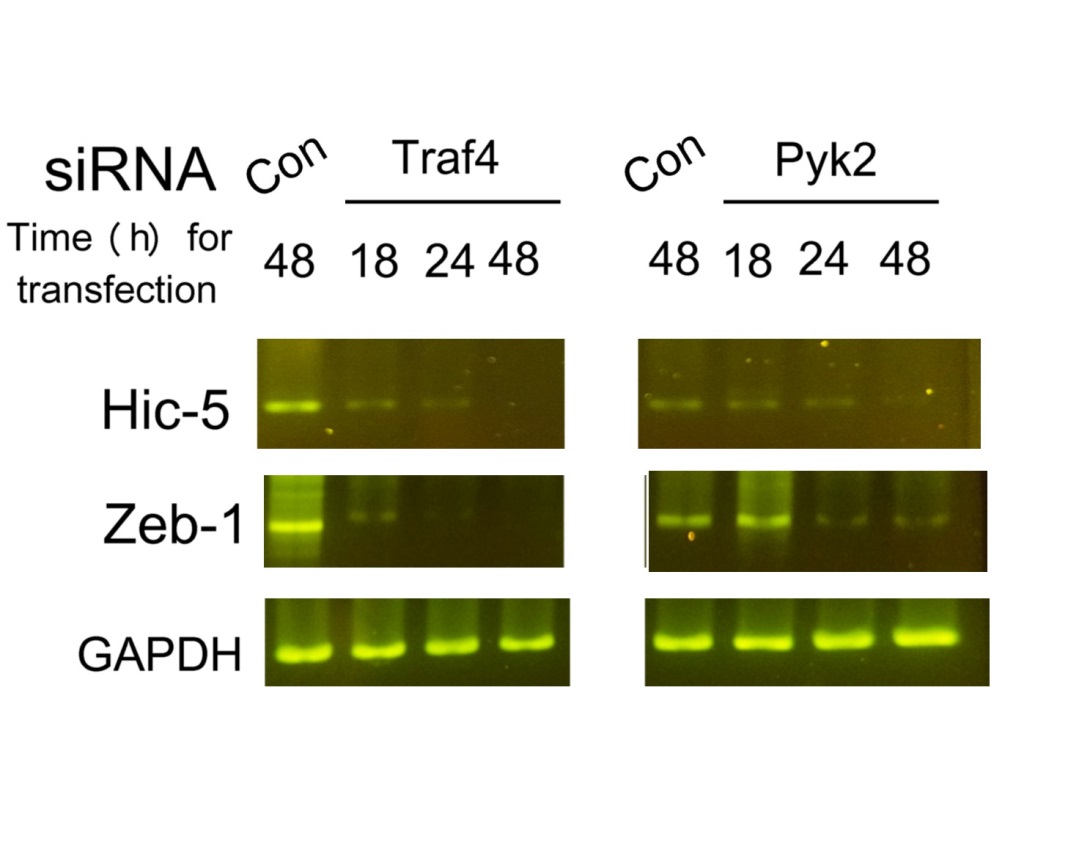


**C.**


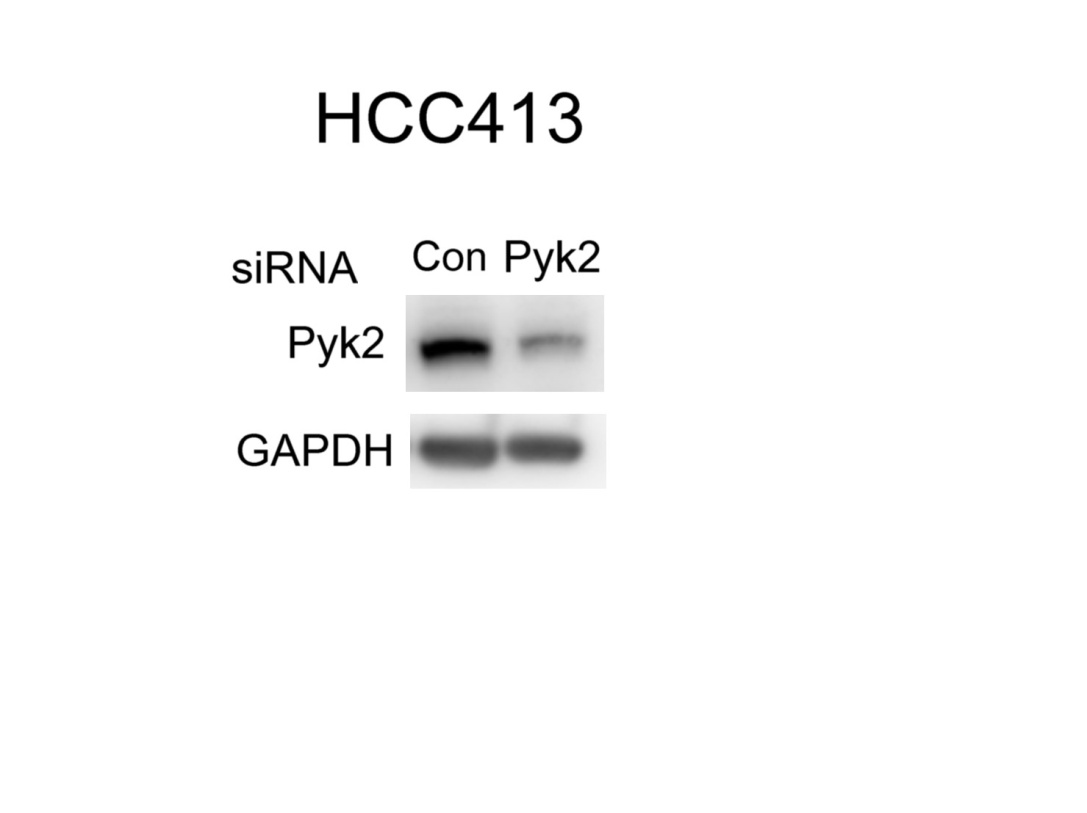


(A) HCC413 cells were co-transfected with p-CMV and control (Con) siRNA or p-Hic-5 coupled with control (Con) or indicated siRNAs. NADPH oxidase activity assays were performed. Relative NADPH oxidase activities were calculated, taking the p-CMV/control siRNA group as 1.0. (*) represent the statistically significant difference (p=0.05, N=3) between each of the indicated samples and (p-Hic-5/control siRNA) group.

(B) HCC413 cells were transfected with control siRNA for 48 h (left and right panel), Traf4 siRNA (left panel) or Pyk2 siRNA (right panel) for 18-48 h. RT-PCRs of Hic-5 and Zeb-1 were performed, using GAPDH as an internal control.

(C) HCC413 cells were transfected with control siRNA (Con) or Pyk2 siRNA for 48 h. Western blot of Pyk2 was performed using GAPDH as an internal control.

The data shown in (B) (C)were representatives of two reproducible results.
