# Supplementary material for: Hydrogen peroxide inducible clone-5 sustains NADPH oxidase-dependent reactive oxygen species-c-jun N-terminal kinase signaling in hepatocellular carcinoma
Source: Oncogenesis. 2019 Aug 6;8(8):40. doi: 10.1038/s41389-019-0149-8 (PMC6684519; doi:10.1038/s41389-019-0149-8)
Supplement: Supplementary file 6 — Supplemental Fig 6 [file 41389_2019_149_MOESM6_ESM.docx]

**Supplemental Fig. 6 Inhibitors of ROS-JNK pathway suppressed constitutive and HGF-induced Hic-5 promoter activity**

(A)


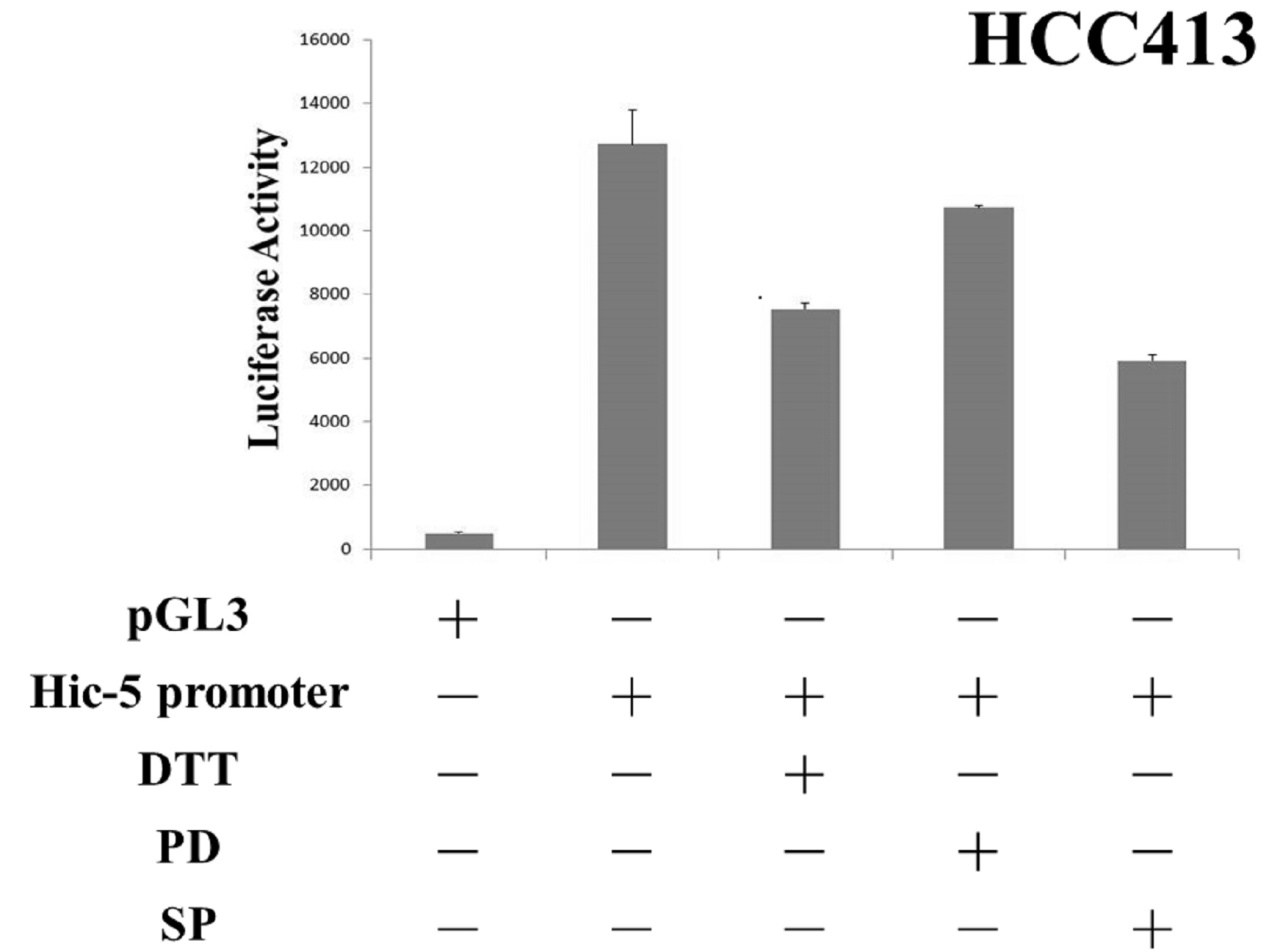


(B)


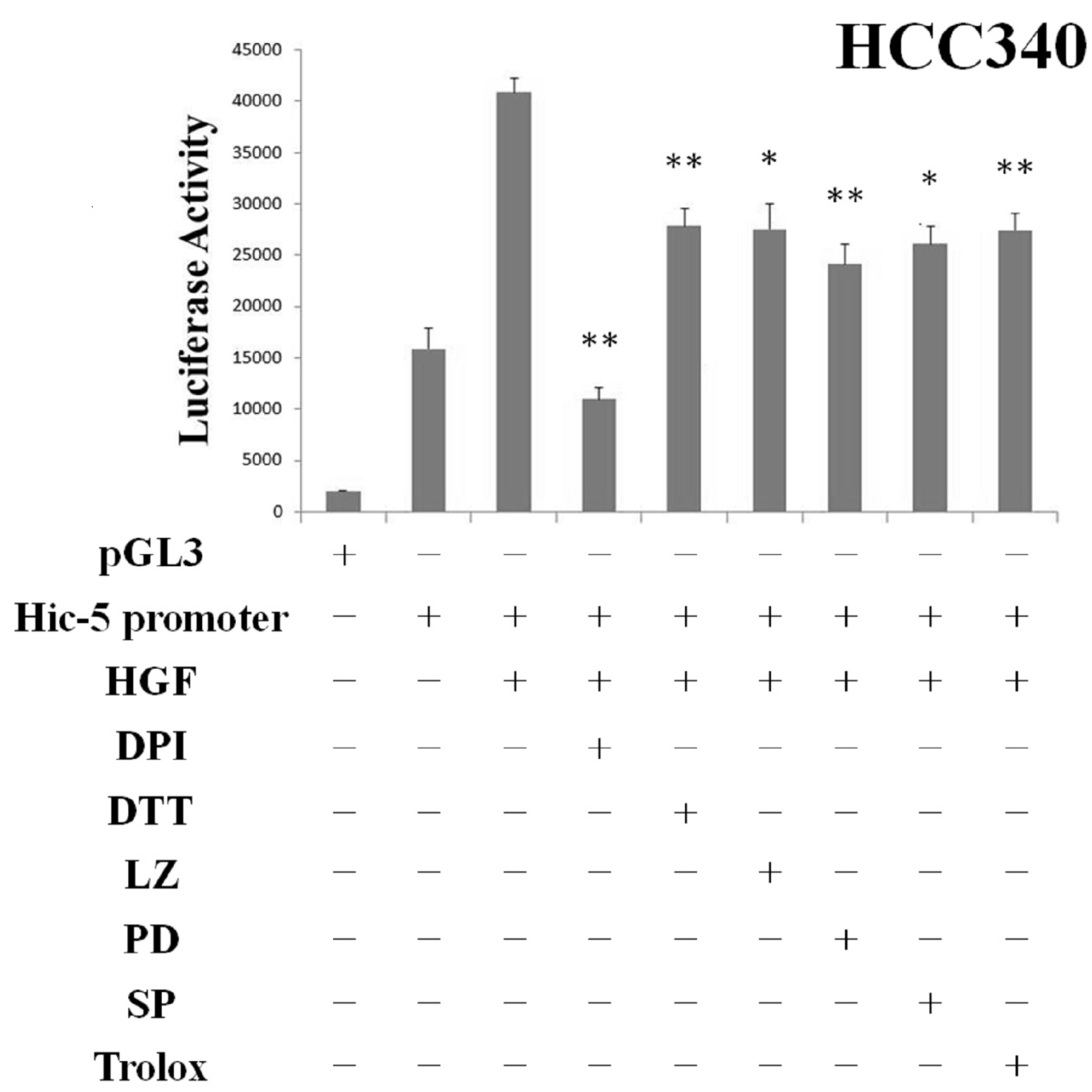


HCC413 (A) and HCC340 (B) were transfected with pGL3 vector or full length Hic-5 promoter, ProHic-5 (1126), for 16 h. The cells transfected with ProHic-5(1126) were untreated or treated with indicated inhibitors for 24 h (A); untreated or treated with HGF or HGF with indicated inhibitors for 24 h (B). Luciferase assay was performed. In (A), the data of luciferase activity was average of two reproducible experiments. In (B), (*, **) represent statistically significance as (p<0.05, p<0.005, n=4) between the indicated sample *vs* the HGF-treated only group.
